# CBX4 suppresses CD8^+^ T cell antitumor immunity by reprogramming glycolytic metabolism

**DOI:** 10.7150/thno.95748

**Published:** 2024-06-17

**Authors:** Jingzeng Wang, Wenlong Jia, Xi zhou, Zhibo Ma, Jing Liu, Peixiang Lan

**Affiliations:** 1 Institute of Organ Transplantation, Tongji Hospital, Tongji Medical College, Huazhong University of Science and Technology, Key Laboratory of Organ Transplantation, Ministry of Education, NHC Key Laboratory of Organ Transplantation, Key Laboratory of Organ Transplantation, Chinese Academy of Medical Sciences, China;; 2 Hepatic Surgery Center, Tongji Hospital, Tongji Medical College, Huazhong University of Science and Technology, Wuhan, China;; 3 Clinical Medical Research Center of Hepatic Surgery at Hubei Province, Wuhan, China.

**Keywords:** CBX4, CD8^+^ T cell, Aldob, glucose metabolism, anti-tumor

## Abstract

**Rationale**: CD8^+^ T cells undergo a series of metabolic reprogramming processes during their activation and proliferation, including increased glycolysis, decreased aerobic oxidation of sugars, increased amino acid metabolism and increased protein synthesis. However, it is still unclear what factors regulate these metabolic reprogramming processes in CD8^+^ T cells in the tumor immune microenvironment.

**Methods**: T cell chromobox protein 4 (CBX4) knock-out mice models were used to determine the role of CBX4 in CD8^+^ T cells on the tumor immune microenvironment and tumor progression. Flow cytometry, Cut-Tag qPCR, Chip-seq, immunoprecipitation, metabolite detection, lentivirus infection and adoptive T cells transfer were performed to explore the underlying mechanisms of CBX4 knock-out in promoting CD8+ T cell activation and inhibiting tumor growth.

**Results**: We found that CBX4 expression was induced in tumor-infiltrating CD8^+^ T cells and inhibited CD8^+^ T cell function by regulating glucose metabolism in tumor tissue. Mechanistically, CBX4 increases the expression of the metabolism-associated molecule aldolase B (Aldob) through sumoylation of trans-acting transcription factor 1 (SP1) and Krüppel-like factor 3 (KLF3). In addition, Aldob inhibits glycolysis and ATP synthesis in T cells by reducing the phosphorylation of the serine/threonine protein kinase (Akt) and ultimately suppresses CD8^+^ T cell function. Significantly, knocking out CBX4 may improve the efficacy of anti-PD-1 therapy by enhancing the function of CD8^+^ T cells in the tumor microenvironment.

**Conclusion**: CBX4 is involved in CD8^+^ T cell metabolic reprogramming and functional persistence in tumor tissues, and serves as an inhibitor in CD8^+^ T cells' glycolysis and effector function.

## Introduction

During their activation and proliferation, T cells undergo metabolic reprogramming from sustainable mitochondrial oxidative phosphorylation (OXPHOS) to intense glycolysis to meet the increased bioenergetic demand 1,2. The switch to glycolysis is directly linked to increased effector function and is thought to occur via T cell receptor (TCR)-linked, phosphoinositide 3-kinase (PI3K) and Akt-mediated mechanistic target of rapamycin (mTOR) complex signaling 3. However, in chronic disease processes such as viral infections and tumors, TCR signaling in response to sustained antigenic stimulation drives T cells into a state of exhaustion with concomitant reductions in glycolysis and energy synthesis 4,5. The exhaustion state induced by prolonged exposure to antigens is characterized by disrupted metabolism, reduced effector function and high expression of immunosuppressive molecules, such as programmed cell death protein 1 (PD1) and hepatitis A virus cellular receptor 2 (Havcr2/Tim-3). PD-1 interacts with programmed death ligand (PD-L1) to provide negative regulatory signals to CD8^+^ T cells via the protein tyrosine phosphatase Src homology region 2-containing tyrosine phosphatase (SHP2). This interaction inhibits Ras-like protein (RAS) and PI3K signaling activated by TCR and CD28 stimulation 6,7. Following these signaling changes, glycolysis and metabolism are impaired in activated T cells, as is the ability of these cells to produce inflammatory cytokines such as interferon-gamma (IFN-γ) and tumor necrosis factor-alpha (TNF-α) and cytotoxic factors such as granzyme B (Gzmb) and perforin 8. The development of strategies to restore a functional metabolic state in exhausted T cells remains an unsolved and difficult problem in tumor immunotherapy.

Polycomb group (PcG) proteins are conserved complexes that have similar roles in maintaining gene repression by modifying chromatin structure 9,10. As a component of the canonical polycomb repressive complex 1 (cPRC1), CBX4 participates in the process by which cPRC1 compacts nucleosome arrays and blocks chromatin remodeling by the mammalian switch/sucrose non-fermentable (mSWI/SNF) complex (also called BAF) 11,12. In addition to its involvement in chromatin landscape and transcriptional control, CBX4 possesses sumoylation activity that endows it with additional protein-interacting functions 13. CBX4 has been reported to function as either an oncogene or a cell proliferation suppressor, depending on its interaction target and the cell line 14. For example, depletion of CBX4 reduces the levels of proliferating cell nuclear antigen (PCNA) and cyclin E2/CCNE2 while increasing the level of cyclin-dependent kinase inhibitor 2A (CDKN2A/p16) to delay the G1/S transition in hepatocellular carcinoma cells 15. Although CBX4 has been identified as essential in tumor cells and stem cells, there have been few reports on its role in immune cells 16.

Our study revealed that knockout of CBX4 in CD8^+^ T cells in tumor tissues promotes intracellular glycolysis and ATP synthesis, ultimately enhancing CD8^+^ T cell antitumor capacity. Specifically, we found that CBX4 promotes the transcription of Aldob, a key enzyme in glucose metabolism, by enhancing the stability of the transcription factor KLF3 and SP1 via sumoylation. Aldob-mediated metabolic reprogramming in CD8^+^ T cells was associated with PI3K-Akt signaling activation. Specific targeting of CBX4 within CD8^+^ T cells effectively synergizes with anti-PD1 therapy. Thus, our data revealed that CBX4 was involved in regulating the metabolism and effector functions of CD8^+^ T cells in the tumor immune microenvironment.

## Materials and methods

### Animals

Wild-type C57BL/6 mice were purchased from Beijing Vital River Laboratory Animal Technology Co., Ltd. *Cbx4^fl/f^*^l^ and *Cd4-cre* mice were donated by the Laboratory of Cardiology, Tongji Hospital of Science and Technology, Huazhong University. *Rag^-/-^* mice and OT-I mice were donated by the Laboratory of Urology, Tongji Hospital of Science and Technology, Huazhong University. All the mice were feeding in SPF animal laboratory. All the mice used in the experiments were executed under anesthesia or painlessly in accordance with the animal ethics institution guidelines of Tongji Hospital. The ethics approval number was TJH-202008014.

### Antibodies and reagents

The rabbit anti-CBX4 antibody (ab242149) was purchased from Abcam, and the rabbit anti-Aldob, rabbit anti-Akt, rabbit anti-pT308-Akt, and rabbit anti-pS473-Akt antibodies were purchased from ABclonal. The Mouse anti-SP1antibody (sc420) and Mouse anti-KLF3 antibody (sc-514500) were purchased from Santacruz. The mouse anti-Flag, mouse anti-Gadph, rabbit anti-Flag, and rabbit anti-Sumo1 antibodies were purchased from Proteintech. The PE anti-CD8, BV510 anti-CD8, BV605 anti-CD8, PE anti-CD4, APC anti-CD4, BV785 anti-CD45, PE/cy5.5 anti-CD134, APC anti-CD44, FITC anti-CD27, APC anti-CD28, PE anti-IFN-γ, FITC anti-TNF-α, BV421 anti-PD1, AF647 anti-Tim3, PE anti-Perforin antibodies and CD3/CD28 antibodies were purchased from Biolegend. Anti-CD4 (BE0003) and anti-CD8 (BE0061) were purchased from BioXcell. DHAP was purchased from Sigma. MK2206 was purchased from MedChemExpress. DMEM and RPMI 1640 cell culture medium was purchased from Gibco. Protein A/G beads were purchased from Beyotime Biotechnology. Fetal bovine serum was purchased from HyClone. The 173-venus-P53 and 174-venus-T vectors were donated, and the 173-venus-*Klf3*, 173-venus-*Sp1*, 173-venus-Ets1, 173-venus-Elf1, 174-venus-*Cbx4*, pCMV-*Klf3*, pCMV-*Sp1*, and Pcagas-*Cbx4*, pCDH-sh-*Aldob* vectors were constructed in our laboratory.

### Cell isolation

(1) Acquisition of splenic CD8^+^ T cells: Mouse spleens were excised, crushed, and resuspended in PBS. After filtration through a 70 µm sterile strainer, the tissue lysate was centrifuged with Ficoll medium at 650 × g for 30 minutes with acceleration and deceleration at 0 × g. After erythrocyte lysis, the cells were incubated with a PE-conjugated anti-CD8 antibody and PE-conjugated magnetic microbeads for 15 minutes. Finally, cells were isolated using a magnetic sorting column, resuspended and cultured in RPMI 1640 medium containing 10% fetal bovine serum and 5 μg/mL anti-CD3/CD28 antibodies. The isolation efficiencies are shown in the supplementary figures (Figure S3B). All the above procedures were performed under sterile conditions. Cells were cultured for 3 days and harvested.

(2) Acquisition of tumor-infiltrating CD8^+^ T cells: After mice were sacrificed, we separated the tumors and ground them to prepare a suspension with PBS. After filtration through a 70 µm sterile sieve, the tumor extracts were centrifuged at 300 × g for 5 min, resuspended in 35% Percoll medium and centrifuged at 650 × g for 30 min with acceleration and deceleration at 0 × g. After erythrocyte lysis, the cells were incubated with a PE-conjugated anti-CD8 antibody and PE-conjugated magnetic microbeads for 15 minutes. Finally, cells were isolated using a magnetic sorting column and used for further experiments. The isolation efficiency is shown in the supplementary figures (Figure S3B).

### Cell culture

(1) 3T3 cells were donated by the Laboratory of Cardiology of Tongji Hospital Science and Technology of Huazhong University, and fetal bovine serum containing 10% DMSO was used to freeze the cells in liquid nitrogen at -110 ℃ for later use. The 3T3 cells were cultured with DMEM containing 10% fetal bovine serum, harvested 48 hours after plasmid transfection when they covered 80 to 90% of the total surface area of the dish, with replacement with fresh medium every other day.

(2) B16F1, B16-OVA and MC38 tumor cells were cultured with DMEM containing 10% fetal bovine serum, harvested when they covered 80 to 90% of the total surface area of the dish, with replacement with fresh medium every other day.

### Intratumoral administration and *in vivo* efficacy experiments

C57BL/6 or *Rag-/-* mice were subcutaneously implanted with 8×10^5^ B16F1/B16-OVA or MC38 cells in a 100 μL volume. The tumor volume and mice weight were measured during two weeks of treatment (length × width^2^/2). At the end of treatment, the tumor tissues were peeled for photographing and weighing.

### Immunoprecipitation and immunoblot analysis

Total protein was extracted from cells lysed using IP lysis buffer (Beyotime, Shanghai, China) containing protease inhibitors. To remove debris, lysates were centrifuged at 12,000×g for 15 min. A two-step IP protocol was then used, with primary antibody incubation overnight followed by incubation with Protein A/G beads for 4 hours. The beads were then washed 3 times with PBS. A microplate reader (BioTek) and BCA protein assay kit (Beyotime, Shanghai, China) were used to measure protein concentrations. Proteins in the samples were separated by electrophoresis on 10% sodium dodecyl sulfate‒polyacrylamide gels, and the proteins were transferred to polyvinylidene fluoride membranes (Millipore, 0.22 μm). The polyvinylidene fluoride membranes were blocked with 5% skim milk prepared with TBST solution and incubated with the primary antibodies for 16 hours.

### Quantitative PCR analysis

A Total RNA Rapid Extraction Kit (Fastagen, Shanghai, China) was used to isolate total mRNA from BMDMs, and a high-capacity cDNA Reverse Transcription Kit (Takara, Shiga, Japan) was used to reverse transcribe total mRNA into cDNA according to the manufacturer's instructions. The SYBR Green kit (Vazyme) was then used to quantify the expression levels of the target genes by quantitative real-time polymerase chain reaction (qRT-PCR) analysis. StepOne software (Thermo Fisher Scientific) was used to analyze the data. The primers were as follows:

*Cd69*: forward, 5-CCCTTGGGCTGTGTTAATAGTG-3; reverse, 5-AACTTCTCGTACAAGCCTGGG-3; *Gitr*: forward, 5-GCCATGCTGTATGGAGTCTCG-3; reverse, 5-CCACTTCCGTTCTGAACCTTG-3; *Cbx4*: forward, 5-AAGAAGCGGATACGCAAGGG-3; reverse, 5-GGAGGAGTCTTGAAGCCCAG-3; *Ifn-γ*: forward, 5-ATGAACGCTACACACTGCATC-3; reverse, 5-CCATCCTTTTGCCAGTTCCTC-3; *Aldob*: forward, 5-GAAACCGCCTGCAAAGGATAA-3; reverse, 5-GAGGGTCTCGTGGAAAAGGAT-3; *Pdcd1*: forward, 5-ACCCTGGTCATTCACTTGGG-3; reverse, 5-CATTTGCTCCCTCTGACACTG-3; *Havcr2*: forward, 5-TCAGGTCTTACCCTCAACTGTG-3; reverse, 5-TCAGGTCTTACCCTCAACTGTG-3; *Tox*: forward, 5-TCAGGTCTTACCCTCAACTGTG-3; reverse, 5-TCCCAATCTCTTGCATCACAGA-3; *Aldob-PR1*: forward, 5-GGGGACAATGTGTGGGGAAA-3; reverse, 5-TCTCTTTCTACCGCAGGGGA-3; *Aldob-PR2*: forward, 5-TGCAGCAAGTGGCTACTGAA-3; reverse, 5-TCACAAGAGCACTGGGTTCG-3; *Aldob-PR3*: forward, 5-TGCCACTATGTCCTTCGTTC-3; reverse, 5-AAATATCAATACCAGACATG-3.

### Vector construction

The pEGFP-n1-venus-173 and pEGFP-n1-venus-174 vectors were digested with EcoRⅠ and AgeⅠ (Thermo Fisher) endonucleases and ligated to PCR-amplified *Klf3, Sp1* or *Cbx4* cDNA sequences with T4 DNA ligase (Thermo Fisher). pCMV-flag vectors were digested with BamHⅠ (Thermo Fisher) and NotⅠ endonucleases and ligated to PCR-amplified *Klf3* and *Sp1* cDNA sequences using homologous recombination DNA ligase (Vazyme). Pcaggs vectors were digested with XhoI and NotⅠ (Thermo Fisher) endonucleases and ligated to PCR-amplified *Cbx4* cDNA sequences using T4 DNA ligase. pCDH vectors were digested with XhoI and EcoRⅠ (Thermo Fisher) endonucleases and ligated to primers of sh-Aldob1/2/3 sequences using T4 DNA ligase. The ligated vectors were then transformed into competent cells, and transformants were selected through antibiotic selection on LB agar and sent for DNA sequencing. After confirmation by sequencing, we amplified the correct colonies and extracted the plasmids using plasmid extraction kits (Omega). The primers used for PCR amplification of the target genes were as follows:

Pcaggs-*Cbx4*: forward, 5-TCGGCGGCCGCatggagctgccagctgtt-3; reverse, 5-TCTGCTAGCTTAcaccgtcacgtattcctt-3; pEGFP-n1-venus-173-*Klf3*: forward, 5-CCGGAATTCatgctcatgtttgatcca-3; reverse, 5-acgACCGGTGGctagcatgtggcgtttcct-3; pEGFP-n1-venus-173-*Sp1*: forward, 5-CCGGAATTCatgagcgaccaagatcac-3; reverse, 5-acgACCGGTGGaaccattgccactgatatt-3; pEGFP-n1-venus-174-*Cbx4*: forward, 5-CCGGAATTCatggagctgccagctgtt-R; reverse, 5-ccgccgccGACCGGTGGcaccgtcacgtattcctt-3; pCMV-*Klf3*: forward, 5-GGGTTTAAACGGATCCatgctcatgtttgatccagtccc-3; reverse, 5-TAGACTCGAGCGGCCGCactagcatgtggcgtttcct-3; pCMV-*Sp1*: forward, 5-GGGTTTAAACGGATCCatgagcgaccaagatcactcca-3; reverse, 5-TAGACTCGAGCGGCCGCaaaccattgccactgatattaatggact-3. pCDH-sh-Aldob1: forward, 5-CCGGT CCCTGGAAATTAGATAACATTCTCGAGAATGTTATCTAATTTCCAGGGTTTTTG-3; reverse, 5-AATTCAAAAACCCTGGAAATTAGATAACATTCTCGAGAATGTTATCTAATTTCCAGGGA-3; pCDH-sh-Aldob1: forward, 5-CCGGTGTGAGGAGGATGCTACACTTACTCGAGTAAGTGTAGCATCCTCCTCACTTTTTG-3; reverse, 5-AATTCAAAAAGTGAGGAGGATGCTACACTTACTCGAGTAAGTGTAGCATCCTCCTCACA-3; pCDH-sh-Aldob3: forward, 5-CCGGTGCCAGGGAAATCTGTTCAGAACTCGAGTTCTGAACAGATTTCCCTGGCTTTTTG-3; reverse, 5-AATTCAAAAAGCCAGGGAAATCTGTTCAGAACTCGAGTTCTGAACAGATTTCCCTGGCA-3.

### Transfection of 3T3 cells

When the 3T3 cells had grown to cover 60-70% of the total surface area of the dish, we transfected the above vectors into the cells at a concentration of 2 μg using the Lipofectamine 6000 Transfection Kit (Beyotime) according to the manufacturer's transfection protocol. After 6 hours of incubation at 37 °C with 5% CO_2_, the culture medium was replaced with fresh DMEM. After culture for an additional 24 h, the cells were harvested for Western blotting as described above, and the cells transfected with the pEGFP-n1-venus-173-*Klf3* or pEGFP-n1-venus-173-*Sp1* and pEGFP-n1-venus-174-*Cbx4* vectors were prepared for immunofluorescence staining. Staining was observed and images were acquired using an upright fluorescence microscope (Nikon Eclipse C1, Japan).

### Lentivirus construction and infection

The PCDH-shAldob plasmid was co-transfected with PMD and PSPAX2 plasmids into HEK293T cells. All cell culture supernatants were collected at 72h and centrifuged twice at 3500 rpm and then ultracentrifugation at 15,000 g for 1 h. The lentivirus was resuspended in complete medium. After that, the virus suspension was infected with OT-I CD8^+^ T cells, centrifuged at 600g at room temperature for 30 min to increase the efficiency of viral infection, and CD8^+^ T cells were cultured for 48 h before tail vein injection.

### Flow cytometric analysis

The Cytofix/Cytoperm Fixation Permeabilization Kit (BD) was used according to the manufacturer's instructions to permeabilize the cell membrane and evaluate the intracellular phenotype and marker expression. Then, a flow cytometer (BD FACSCelesta) was used to determine the percentages of positive cells. Data were analyzed by using FlowJo software. The strategies of FlowJo analysis are shown in the supplementary figures (Figure S3C, D).

### ELISA

A mouse IFN-γ ELISA kit and a mouse TNF-α ELISA kit (ABclonal) were used according to the manufacturer's instructions. Cell culture supernatant of CD8^+^ T cells with stimulation for 3 days were acquired for ELISA test. A microplate reader (BioTek) was used to measure the OD at 450 nm and calculate the cytokine concentrations.

### Metabolite detection

Metabolite detection kits, such as pyruvate, lactate, α-ketoglutarate, malate, and citrate detection kits, were purchased from Solarbio Life Sciences. ATP detection kits were purchased from Beyotime Biotechnology. All kits were used following the instructions, and a microplate reader (BioTek) was used to measure the OD at different wavelengths and calculate the metabolite concentrations.

### Seahorse analysis

Seahorse kits were purchased from Agilent Technologies, and experiments were conducted according to the instructions. Wave software (Agilent Technologies) was used for analysis of the data.

### Cut-Tag qPCR

The Cut-Tag qPCR experiments were conducted using Hyperactive Universal CUT&Tag Assay Kit from Vazyme (TD904), and experiments were conducted according to the instructions. The qPCR primers were mentioned above with *Aldob-PR1/2/3.*

### Chromatin immunoprecipitation followed by sequencing

Cells were washed in FBS-free medium and then subjected to crosslinking with 1% formaldehyde for 10 min at room temperature. After crosslinking, the cells were washed with ice-cold PBS. For each sample, 20 million fixed cells were lysed to prepare nuclear extracts. After chromatin shearing by sonication, the lysates were incubated overnight at 4 °C with antibody (5 µg)-conjugated protein A Dynabeads. After immunoprecipitation, the beads were recovered using a magnet and washed. DNA was eluted, crosslinking was reversed by incubation at 65 °C for 4 hours, and DNA was then purified with a QIAGEN kit.

DNA was quantified using the Qubit® dsDNA HS assay and a Qubit® 2.0 fluorimeter (Invitrogen). For ChIP-Seq, 5 ng of purified ChIP DNA was used to generate the sequencing library using an NEB kit, and the library was sequenced with the Illumina HiSeq X Ten instrument. Raw sequence reads were first processed by FastQC for quality control, and adapter sequences and poor-quality reads were then removed. Quality-filtered reads were then mapped to the reference genome using BWA or Bowtie, and only uniquely mapped reads were retained. Sam files were converted to Bam format using SAMtools and used for peak calling. MACS2 was used to call peaks with the sonicated input as a control and an initial threshold q-value of 0.01 as the cutoff. Visualization of read count data was performed by converting raw bam files to bigWig files using IGV Tools. BETA or ChIP seeker was used to analyze expression data and binding data 17. ChIP-seq data analysis was performed by DIATRE Biotechnology, Shanghai, China.

### RNA sequencing analysis

After extracting total RNA from the samples, eukaryotic mRNA was enriched with oligo(dT)-conjugated magnetic beads. mRNA was cleaved into short fragments by adding fragmentation buffer. This mRNA was used as a template to synthesize first-strand cDNA using random hexamers, and second-strand cDNA was then synthesized by adding dNTPs, RNase H and DNA polymerase I. Next, the double-stranded cDNA was subjected to end repair, poly(A) tailing, ligation of sequencing junctions, purification and fragment selection using magnetic beads, and PCR amplification was finally conducted to obtain the library. Sequencing was performed after the library passed quality control. We used fastp to filter the raw data and then used FastQC for quality control of the clean data. For experiments with biological duplicates, we used DESeq2 for differential expression analysis, and the p value calculated in the differential expression analysis was corrected for multiple hypothesis testing by controlling the FDR (false discovery rate) to determine the threshold p value. In our analysis, differentially expressed genes in biological duplicates were defined as those with |FoldChange| ≥ 2 and padj ≤ 0.05. Based on the results of the differential expression analysis and GO annotations, we performed GO enrichment analysis using clusterProfiler with p-adjust (FDR) < 0.05 as the threshold for filtering for significant enrichment. GO terms that met this criterion were defined as significantly enriched in the differentially expressed genes. Similar to the GO enrichment analysis process, we used clusterProfiler based on the results of differential expression analysis and KEGG annotations to identify significantly enriched KEGG pathways using p-adjust (FDR) < 0.05 as the threshold 18-20. RNA-seq data analysis was performed by Bioyigene Biotechnology, Wuhan, China.

### Single-cell RNA sequencing analysis

The single-cell RNA-seq data were downloaded from GSE148673 21. The Seurat package (v.4.4.0) was applied for processing of the gene expression matrix 22. Specifically, the expression data were normalized using the NormalizeData function with default parameters. The FindVariableFeatures function with the vst method was used to filter the top 3000 variable genes. To correct for confounding factors, anchor-based correction was conducted to merge samples across patients. Termed “anchors” were calculated based on the most variable genes using the FindIntegrationAnchors function. The expression levels of these variable genes in each sample were corrected using the generated anchors and combined into a single Seurat object by the IntegrateData function with 30 dimensions used in the anchor weighting procedure 23. Principal component analysis (PCA) and uniform manifold approximation and projection (UMAP) analysis were performed for dimensionality reduction of gene expression data. To explore the expression profile of *Cbx4* in diverse cell types, the expression of *Cbx4* was visualized using the Seurat and ComplexHeatmap packages.

### Statistical analysis

All statistical analyses were conducted with Prism software (GraphPad). All data are expressed as the means±SD. One-way ANOVA was used for comparisons among multiple groups, and an unpaired t test was used for comparisons between 2 groups. P values <0.05 were considered to indicate statistically significant differences. * P <0.05; ** P<0.01; *** P<0.001; **** P<0.0001; ns represents no statistical significance.

## Results

### Anti-tumor immune responses are improved in cKO (*Cbx4^fl/fl^Cd4-cre*) mice

**Figure 1:**
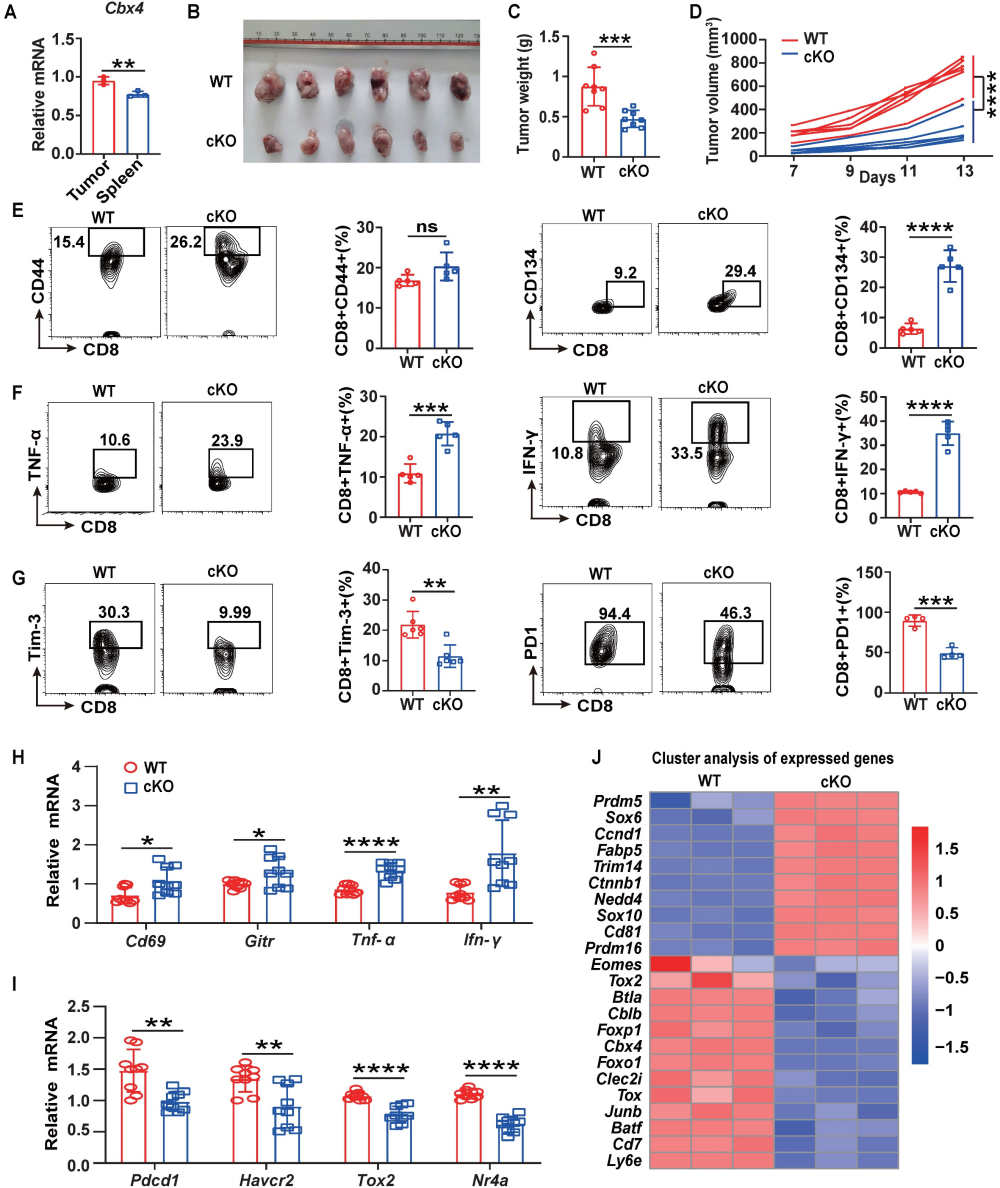
***Cbx4* knockout T cells exhibit enhanced antitumor function.** (A) RT-PCR analysis of *Cbx4* expression in splenic and tumor CD8^+^ T cells. (B-D) B16F1 tumor growth (B), volume (C) and weight (D) in cKO and WT mice on Day 14 after cell inoculation. (E-G) Flow cytometric analysis results showing the percentages of CD44^+^ and CD134^+^ (E), TNF-α^+^ and IFN-γ^+^ (F) and PD1^+^ and Tim3^+^ (G) CD8^+^ T cells in subcutaneous B16F1 tumors of WT or cKO mice 14 days after tumor cell inoculation. (H, I) RT-PCR analysis of effector T cell (H) and exhausted T cell (I) associated gene expression in B16F1 tumor CD8^+^ T cells from WT and cKO mice. (J) Gene signature analysis of WT and cKO B16F1 tumor CD8^+^ T cells. n=3 mice per group (A); n=6 mice per group (B, C and D); n=5 mice per group (E and F); Tim3^+^, n=6 mice per group, PD1^+^, n=4 mice per group (G); n=9 mice per group (H, I). The data are representative of three independent experiments (A-I). Statistical significance was determined by two-tailed unpaired Student's t test (A-I). The data are presented as the means ± SEM. * P <0.05; ** P<0.01; *** P<0.001; **** P<0.0001; ns represents no statistical significance.

Knockout of *Cbx4* in human MSCs results in destabilised nucleolar heterochromatin, enhanced ribosome biogenesis, increased protein translation and accelerated cellular senescence, so does CBX4 also affect the function of immune cells 24? To answer this, we first analyzed human tumor single-cell RNA sequencing datasets from GEO database and found that *Cbx4* were highly expressed in tumor exhausted T cells (Tex) (Figure S1A-C). High expression of *Cbx4* is also tightly associated with shorter years of tumor survival in patients (Figure S1D-F). In view of these results, we examined the expression of *Cbx4* in mouse tumor (Relative exhausted subsets) and splenic (Relative naïve subsets) CD8^+^ T cells and found that *Cbx4* was more highly expressed in tumor CD8^+^ T cells (Figure 1A). Next, we produced T cell-specific *Cbx4*-knockout mice (*Cbx4^fl/fl^-Cd4cre*, cKO mice) to investigate function of CBX4 in exhausted T cells (Figure S2A-C). We detected percentages of CD4^+^ T cells and CD8^+^ T cells in thymus, spleen and blood in cKO mice. And there were no significant differences, suggesting that CBX4 has no effect on CD4^+^ and CD8^+^ T cell development. (Figure S2D). Then by establishing B16F1 tumor-bearing models in cKO mice and wild-type (WT) mice, we found that knockout of CBX4 in T cells significantly inhibited tumor growth (Figure 1B). Consistently, tumor volume and weight were also reduced in cKO mice (Figure 1C, D). Next, we examined the alteration of T cells in the tumor tissue of two different mice by flow assay. The proportion of CD8^+^ T cells did not change in cKO mice compared to WT mice (Figure S3B), suggesting that CBX4 may not affect the proliferative activity of T cells. However, the percentage of CD44^+^ CD8^+^ and CD134^+^ CD8^+^ T cells in tumor were increased in cKO mice compared to WT mice (Figure 1E). Increased production of IFN-γ and TNF-α was also observed in cKO mice (Figure 1F). This phenomenon showed that knocking out *Cbx4* in T cells could enhance the activation and effector capacity of T cells in CD8^+^ subsets. Besides, the proportions of PD1^+^ and Tim3^+^ cells among CD8^+^ T cells in tumor were diminished in cKO mice (Figure 1G). These results suggest that knockout of Cbx4 enhances the activation of tumor-infiltrated CD8+ T cells thus reinforcing the anti-tumor immunity.

To further investigate the role of CBX4 in T cells, we isolated CD8^+^ T cells from tumors in the B16F1 tumor-bearing model on day 14, and quantitative PCR (qPCR) was performed to measure gene expression. Consistent with the flow cytometric analysis results, the expression of activator molecules, including *Cd69* and *Gitr*, and effector molecules, including *Ifn-γ* and *Tnf-α*, was increased in CD8^+^ T cells in the tumors of cKO mice (Figure 1H). In contrast, the expression of immunoinhibitory factors such as *Pdcd1* and *Hacvr2*, as well as transcription factors that promote exhaustion, including *Tox1* and *NR4a*, was downregulated in the CD8^+^ T cells in the tumors of cKO mice (Figure 1I). Moreover, through RNA sequencing analysis in tumor CD8^+^ T cells, we found that genes associated with effector functions, such as *Sox6*, *Ccnd1*, *CD81* and *Trim14,* were upregulated, while genes associated with immune inhibition, such as *Tox*, *Jun-b*, *Btla*, *Batf* and *Foxp1* were downregulated (Figure 1J) 25-27. To further conform the function of CD8^+^ T cells, we depleted CD4^+^ T cells or CD8^+^ T cells using anti-CD4 or anti-CD8 antibodies. Our data showed that depletion of CD8+ T cells reversed the anti-tumor effect and inhibited anti-tumor immunity in cKO mice (Fig. S4). These data showed that CBX4 in CD8^+^ T cells exhibit the effect on tumors. To confirm that the effect of CBX4 on CD8^+^ T cells is independent of tumor type, we established an MC38 tumor model and observed the same results (Figure S5A-E). These results suggest that knockout of *Cbx4* can enhance the activation and antitumor effect of CD8^+^ T cells.

**Figure 2:**
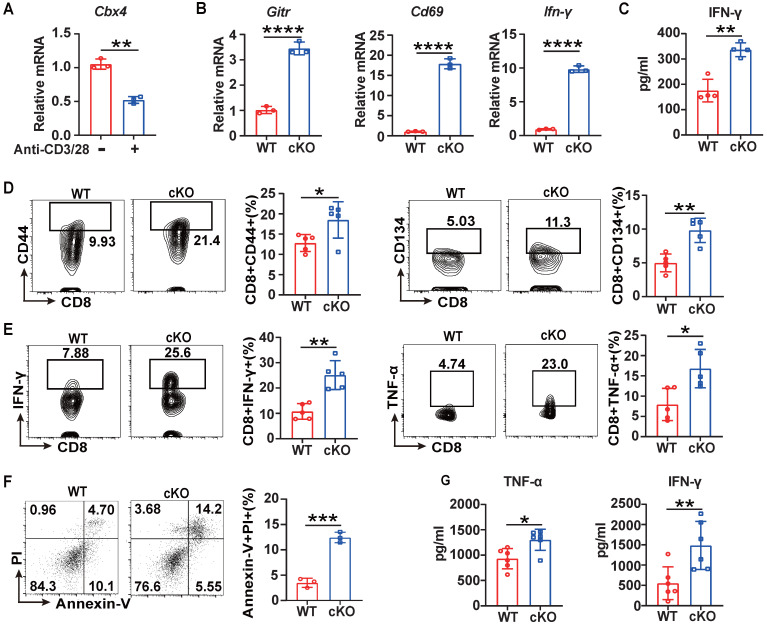
** Knockout of *Cbx4* in T cells facilitated T cell activation and effector functions *in vitro*.** (A) RT-PCR analysis of *Cbx4* expression in naïve and stimulated CD8^+^ T cells. (B) RT-PCR analysis of activation markers in stimulated splenic CD8^+^ T cells from WT and cKO mice. (C) ELISA of IFN-γ in the culture supernatant of cKO and WT splenic CD8^+^ T cells after anti-CD3/CD28 antibody stimulation for 3 days. (D, E) Flow cytometric analysis results showing the percentages of CD44^+^ and CD134^+^ (E) and TNF-α^+^ and IFN-γ^+^ (F) splenic CD8^+^ T cells after anti-CD3/CD28 antibody stimulation for 3 days. (F) Flow cytometric analysis of apoptosis in B16F1 cells after coculture with CD8^+^ T cells at a ratio of 1:5 for 24 h. (G) ELISA of IFN-γ and TNF-α in culture supernatant from B16F1 tumor cells cocultured with CD8^+^ T cells as described above. n=3 mice per group (A and B); n=4 mice per group (C); n=5 mice per group (D, E); n=3 mice per group (F); n=6 per group (G). The data are representative of three independent experiments (A-G). Statistical significance was determined by two-tailed unpaired Student's t test (A-G). The data are presented as the means ± SEM. * P <0.05; ** P<0.01; **** P<0.0001.

For further investigation of CBX4's function in CD8^+^ T cells, we performed short-term acute stimulation of T cells *in vitro*. For the *in vitro* short-term study, splenic CD8^+^ T cells were isolated and stimulated with anti-CD3/CD28 antibodies for three days. *Cbx4* in CD8^+^ T cells gradually decayed after anti-CD3/CD28 antibody stimulation (Figure 2A), similar to the results observed in tumor and splenic T cells (Figure 1A). Next, we investigated the role of CBX4 in T cell proliferation and observed no alterations on proliferation of CD8^+^ T cells in cKO mice after anti-CD3/CD28 antibody stimulation compared with WT mice (Figure S3A). Moreover, in cKO CD8^+^ T cells, the expression of* Cd69*, *Gitr* and *Ifn-γ* was higher than that in WT CD8^+^ T cells at the transcriptional level (Figure 2B, C). The activation of CD8^+^ T cells was also increased after CD3/CD28 stimulation *in vitro* (Figure 2D, E). To further assess the direct activation of CD8^+^ T cell function induced by knockout of *Cbx4*, we cocultured stimulated CD8^+^ T cells with Cell Trace-labeled B16-F1 cells at a ratio of 5:1 for 24 hours. The killing of tumor cells by CD8^+^ T cells was more significant (Figure 2F) and more IFN-γ and TNF-α were produced after co-coculture (Figure 2G). Taken together, these results show that cKO CD8^+^ T cells exhibit superior antitumor capacity and effector functions both upon sustained antigen stimulation and in the early robust activation phase.

### CBX4 inhibits CD8^+^ T cell function by increases the transcription of *Aldob*

**Figure 3:**
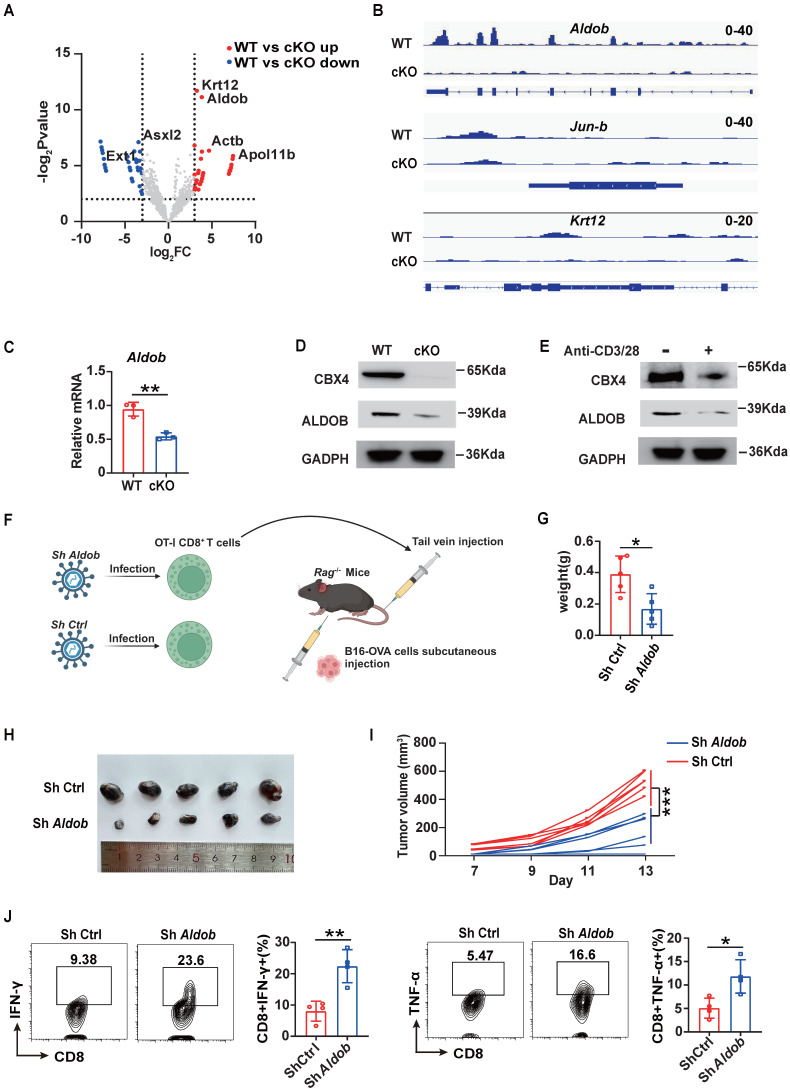
** Knockout of *Cbx4* in CD8^+^ T cells alters the transcription of *Aldob* thus reinforcing CD8^+^ T cells' functions.** (A) Analysis of changed gene loci of CBX4-occupied genes based on the P value and LogFc value. (B) Analysis of CBX4-occupied *Aldob*, *Jun-b*, and *Krt12* gene loci signatures. (C, D) RT-PCR analysis (C) and Immunoblot analysis (D) of *Aldob* in tumor-derived CD8^+^ T cells from WT and cKO mice. (E) Immunoblot analysis of CBX4 and Aldob in naïve and stimulated CD8^+^ T cells. (F) Schematic of virus infectious OT-I CD8^+^ T cells injection and B16-OVA tumor bearing model in *Rag^-/-^* mice. (G-I) B16-OVA tumor weight (G), growth (H) and volume (I) on *Rag^-/-^* mice at day 14. (J) Flow cytometric analysis results showing the percentages of TNF-α^+^ and IFN-γ^+^ CD8^+^ T cells in subcutaneous B16-OVA tumors of *Rag^-/-^* mice. n=3 mice per group (C); n=5 mice per group (G-I); n=4 mice per group (J). The data are representative of three independent experiments (A-I). Statistical significance was determined by two-tailed unpaired Student's t test (D). The data are presented as the means ± SEM. ** P<0.01. ** P<0.1; *** P<0.001.

To further explore the mechanism by which CBX4 inhibits CD8^+^ T cell function, we first performed chromatin immunoprecipitation sequencing (ChIP-seq) of CD8^+^ T cells in cKO and WT tumors using an anti-CBX4 antibody. However, the results revealed that upon knockout of *Cbx4*, there was almost no change in chromatin accessibility (Figure S6A). In terms of the gene loci change, 311 gene loci were reduced following knockout of *Cbx4* (Figure 3A). Among these genes, the loci of *Aldob*, which is tightly associated with glucose metabolism, and the immune inhibitor-associated transcription factor *Jun-b* exhibited the most robust changes, while the *Krt12* locus exhibited only minor variations (Figure 3B). Previous research had reported that CBX4 can inhibit transcription of *Pdcd1* 28. To verify CBX4 whether directly affect expression of *Pdcd1*, we searched the chip data but didn't detect the combination of CBX4 and locus of *Pdcd1*(Figure S6B). In accordance with the ChIP-seq results, knockout of *Cbx4* reduced the production of Aldob at both the transcriptional and translational levels in CD8^+^ T cells in tumors (Figure 3C, D). Upon CD8^+^ T cell activation by CD3/CD28 antibody stimulation, Aldob expression was diminished, accompanied by a decrease in *Cbx4* expression (Figure 3E). To conform the role of Aldob in CD8+ T cell-mediated anti-tumor immunity in cKO mice, we knocked down *Aldob* in OT-I CD8+ T cells (Figure S7 A, B). OT-I CD8^+^ T cells infected with sh-Aldob lentivirus and control virus were then transfused into *Rag^-/-^* mice by tail vein injection, and B16-OVA tumor cells were used for subcutaneous tumor-bearing at the same time (Figure 3F). The results showed that the volume and weight of subcutaneous tumors in *Rag^-/-^* mice decreased significantly after knocking down the *Aldob* expression of OT-I CD8^+^ T cells compared with the control-virus infection group (Figure 3G-I). Besides, we also detected functions of CD8^+^ T cells in tumor tissues and found that knockdown of *Aldob* facilitated effector functions of CD8^+^ T cells (Figure 3J). This data showed that CBX4-Aldob axis sin CD8^+^ T cells exhibit the effect on tumors.

### CBX4 boosts the transcription of *Aldob* by promoting the stability of KLF3 and SP1

**Figure 4:**
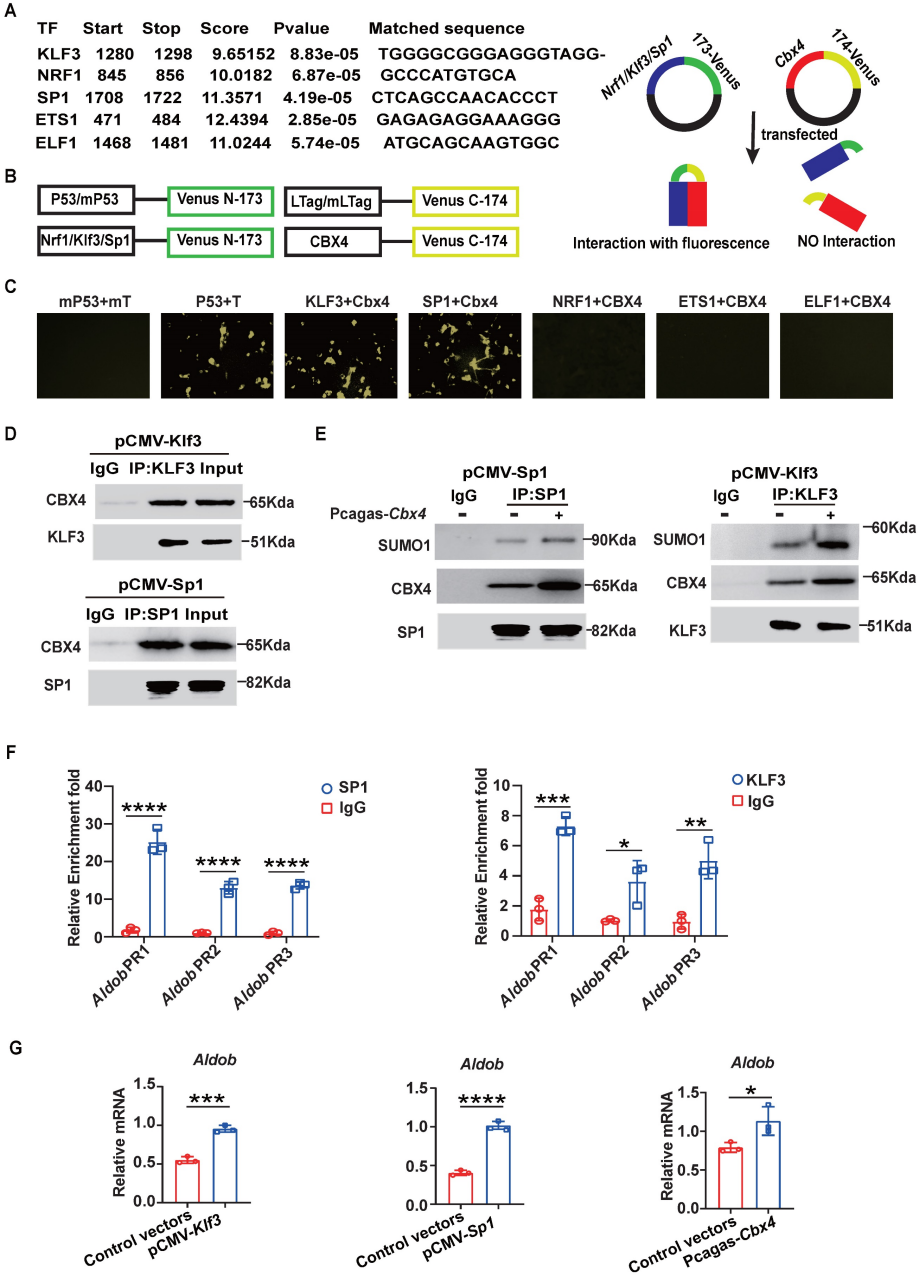
** CBX4 increases *Aldob* expression by promoting the stability and function of KLF3 and SP1.** (A) Prediction of transcription factors for *Aldob* based on AnimalTFDB v4.0. (B) Schematic of the bimolecular fluorescence complementation assay. (C) Immunofluorescence colocalization analysis of CBX4 with SP1 and KLF3 in 3T3 cells after transfection. (D) Immunoblot analysis of CBX4, SP1 and KLF3 after immunoprecipitation with an anti-SP1 or anti-KLF3 antibody in 3T3 cells transfected with vectors. (E) Immunoblot analysis of SUMO-1, CBX4 and SP1 or KLF3 after immunoprecipitation with an anti-SP1 or anti-KLF3 in 3T3 cells transfected with vectors. (F) CUT-Tag qPCR results of spleen CD8^+^ T cells after SP1 or KLF3 immunoprecipitation. (G) RT-PCR analysis of *Aldob* in 3T3 cells after transfection with Pcagas-*Cbx4*, pCMV-*Klf3* or pCMV-*Sp1* vectors. n=3 per group (F, G). Statistical significance was determined by two-tailed unpaired Student's t test, with P values noted in the figures. The data are from three independent experiments (A-G). The data are presented as the means ± SD. * P <0.05; *** P<0.001; **** P<0.0001. IP, immunoprecipitation. Pcagas-*Cbx4*, *Cbx4*-overexpressing Pcagas vectors; pCMV-*Klf3*/*Sp1*, *Klf3* or *Sp1*-overexpressing Pcmv vectors. *Aldob* PR, *Aldob* promoter region.

The ChIP-seq results revealed that CBX4 bound to the 3'UTR of *Aldob* and intergenic regions of *Jun-b*. However, CBX4 as a chromatin structure does not function as a transcription factor to promote transcription.^27^ So, is CBX4 regulating *Aldob* transcription by sumoylation of other transcription factors? First, we analyzed the motif of CBX4 binding sequences and found that it shares more common gene binding regions with transcription factors (TFs) such as ETS1, NRF1, KLF3, SP1 and ELF1 (Figure S6C). Moreover, using the online tool AnimalTFDB v4.0 to predict transcription factors that bind to the promoter region of Aldob based on the nucleotide sequence, we surprisingly found that the TFs that potentially bind to the promoter region of Aldob were the same as those mentioned above, including ETS1, NRF1, KLF3, SP1 and ELF1 (Figure 4A). Based on the features of CBX4, we proposed the hypothesis that CBX4 interacts with these TFs and promotes their stability through sumoylation, ultimately increasing the transcription of *Aldob*. To verify this hypothesis, we used a bimolecular fluorescence complementation (BiFC) assay to identify the binding TFs of CBX4 (Figure 4B). 3T3 cells cotransfected with *Cbx4*-venus-174 and *Klf3*-venus-173 or *Sp1*-venus-173 exhibited significant fluorescence, confirming the interaction of CBX4 with KLF3 and SP1 (Figure 4C). Furthermore, we confirmed these interactions by immunoprecipitation (Figure 4D). Sumoylation of substrates can contribute to the formation of membraneless nuclear structures, which in turn enhances protein stability, and previous reports have confirmed that CBX4 sumoylates the polycomb repressive complex 2 subunit enhancer of zeste 2 (EZH2) to enhance the H3K27 methyltransferase activity of EZH2 29. To identify whether CBX4 affects the sumoylation of KLF3 and SP1, anti-Sumo1 antibodies were then used to detect the sumoylation of KLF3 and SP1 when CBX4 was overexpressed in 3T3 cells. The results indicated that CBX4 increases the sumoylation of KLF3 and SP1, thereby stabilizing their structures and promoting their functions (Figure 4E). To further define whether Spl and Klf3 are Aldob's transcription factors, we performed CUT-Tag qPCR assay in spleen derived CD8^+^ T cells and confirmed that SP1 and KLF3 bound to the promoters of *Aldob* (Figure 4F). Moreover, CBX4, SP1 and KLF3 all up-regulated the expression of Aldob (Figure 4G). In summary, our results showed that CBX4 regulated the functions of KLF3 and SP1 by promoting their sumoylation, thereby indirectly promoting the expression of Aldob.

### Aldob inhibits CD8^+^ T cell function through effects on cellular metabolism

**Figure 5:**
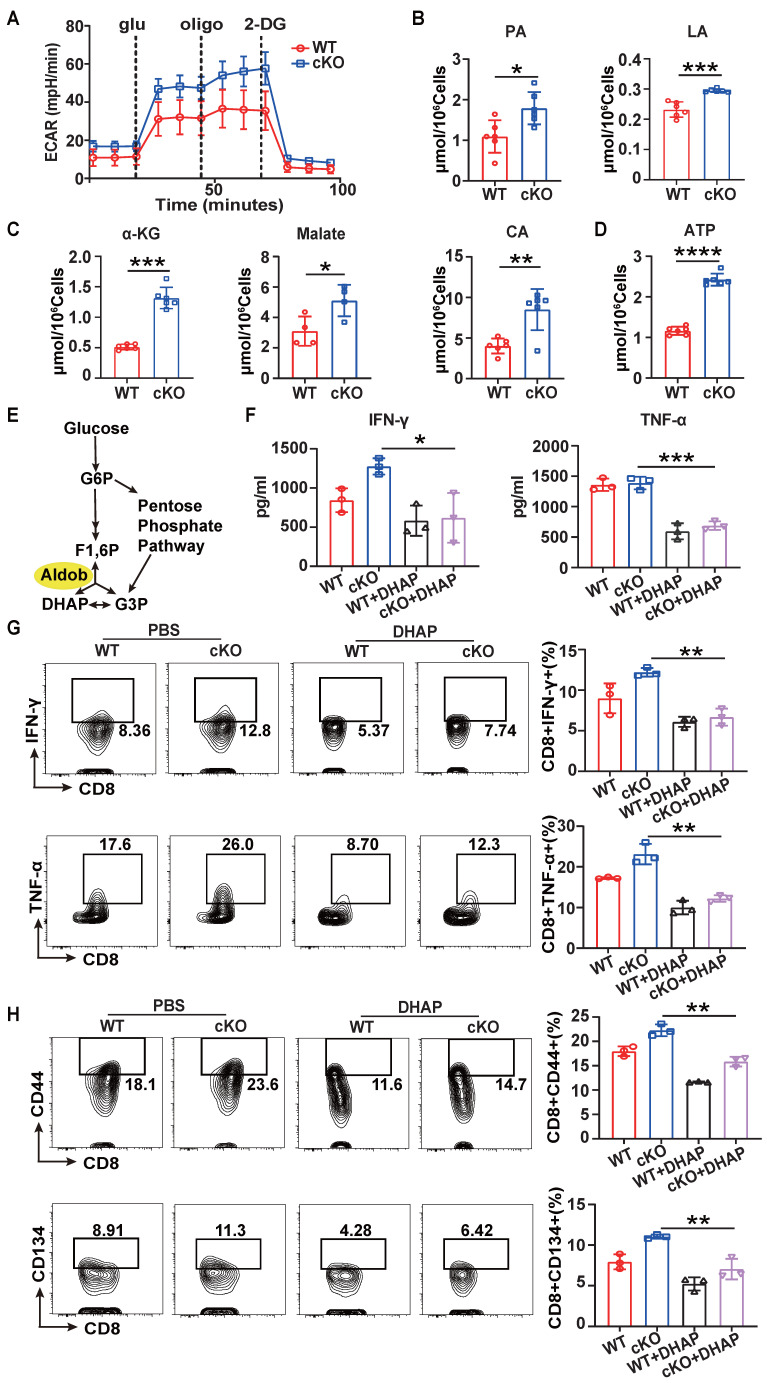
** Aldob represses glycolysis and energy production in T cells.** (A) Seahorse analysis of the ECAR in stimulated WT and cKO splenic CD8^+^ T cells. (B) Measurement of pyruvate and lactate levels in stimulated splenic CD8^+^ T cells. (C, D) Measurement of α-ketoglutarate, malate, citrate (C) and ATP (D) levels in stimulated splenic CD8^+^ T cells. (E) Diagram of the catalytic pathway of Aldob. (F) ELISA of IFN-γ and TNF-α in the culture supernatant of stimulated splenic CD8^+^ T cells with or without the addition of 5 μM DHAP. (G, H) Flow cytometric analysis results showing the percentages of TNF-α^+^ and IFN-γ^+^ (G) and CD44^+^ and CD134^+^ (H) stimulated splenic CD8^+^ T cells with or without DHAP addition. n=6 mice per group (A, B); α-ketoglutarate, citrate and ATP measurement, n=6 mice per group; malate measurement, n=4 mice per group (C, D); n=3 mice per group (F-H). Statistical significance was determined by two-tailed unpaired Student's t test (A-D) and one-way ANOVA followed by Tukey's test (F-H). The data are presented as the means ± SD. * P <0.05; ** P<0.01; *** P<0.001; **** P<0.0001. Glu, glucose; Oli, oligomycin; 2-DG, 2-deoxyglucose; PA, pyruvate; LA, lactate; α-KG, α-ketoglutarate; CA, citrate; G6P, glucose-6-phosphate; F1,6P, fructose-1,6-bisphosphate; G3P, glyceraldehyde-3-phosphate; DHAP, dihydroxyacetone phosphate.

Since Aldob is one of the aldolases involved in glycolysis, we further investigated whether the enhancement of CD8^+^ T cell function induced by knockout of *Cbx4* is related to Aldob-induced alterations in cellular metabolism 30. Seahorse analysis showed a higher extracellular acidification rate (ECAR) in cKO splenic CD8^+^ T cells (Figure 5A) after anti-CD3/CD28 antibody stimulation for 3 days, suggesting that Aldob may serve as a glycolysis inhibitor during T cell activation. Moreover, we found that the quantities of pyruvate and lactate were elevated in cKO splenic CD8^+^ T cells (Figure 5B). Considering that glycolysis is responsible for generating the majority of the energy supply during T cell activation, we subsequently measured the levels of Krebs cycle metabolites and ATP 31. Consistent with the previous results, more Krebs cycle metabolites and ATP were produced in cKO splenic CD8^+^ T cells (Figure 5C, D), illustrating that CBX4 disrupted the glycolysis process and attenuated the subsequent energy production in T cells. Since Aldob reversibly catalyzes the conversion of 1,6-diphosphofructose-fructose (F1,6P) to glyceraldehyde-3-phosphate (G3P) and dihydroxyacetone phosphate (DHAP) (Figure 5E), we utilized Aldob's catalytic product DHAP to confirm Aldob's effects on T cell activation. We observed that 5 μM DHAP inhibited the synthesis and secretion of IFN-γ and TNF-α by splenic CD8^+^ T cells in both cKO and WT mice (Figure 5F, G). Similarly, the activation of CD8^+^ T cells was also reduced by DHAP (Figure 5H). Because CBX4 has been reported to be associated with senescence and metabolism is also tightly connected with senescence, we evaluated the senescence of cKO CD8^+^ T cells 24,32. In cKO mice, the senescence inhibition markers CD27 and CD28 were induced in tumor-infiltrating CD8^+^ T cells (Figure S8A), whereas the level of the metabolism-associated senescent cell marker β-gal was reduced (Figure S8B), suggesting that knockout of *Cbx4* enhances the anti-senescence capacity of T cells. However, upon anti-CD3/CD28 antibody stimulation *in vitro*, no differences were observed in the levels of β-gal, CD27 and CD28 between cKO and WT CD8^+^ T cells (Figure S8C, D), implying that CBX4 is required for senescence during T cell exhaustion but not T cell activation. These data suggest that Aldob-regulated glycolysis and energy metabolism mediate the inhibition of T cell function by CBX4.

### Aldob attenuates T cell metabolism by inhibiting PI3K-Akt signaling

**Figure 6:**
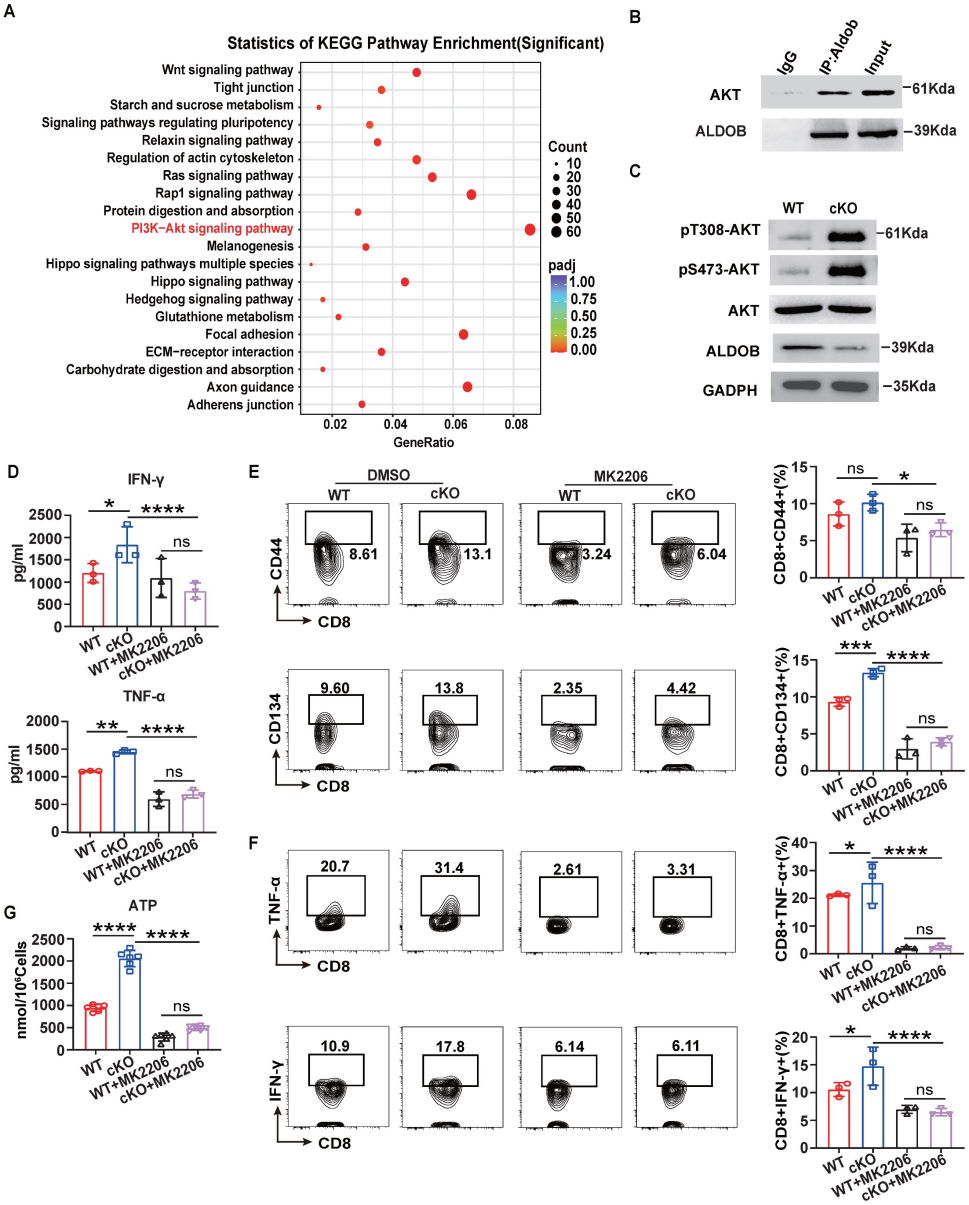
** Akt signaling mediates Aldob-regulated cellular metabolism.** (A) KEGG pathway enrichment analysis of WT and cKO tumor-derived CD8^+^ T cells. (B) Immunoblot analysis of Akt and Aldob after immunoprecipitation with an anti-Aldob antibody in WT tumor CD8^+^ T cells. (C) Immunoblot analysis of pT308-Akt, pS473-Akt, Akt and Aldob in WT and cKO tumor-derived CD8^+^ T cells. (D) ELISA of IFN-γ and TNF-α in culture supernatants from splenic CD8^+^ T cells with or without 1 μM MK-2206 addition. (E, F) Flow cytometric analysis results showing the percentages of CD44^+^ and CD134^+^ (E) and TNF-α^+^ and IFN-γ^+^ (F) stimulated splenic CD8^+^ T cells with or without MK-2206 addition. (G) Measurement of ATP levels in WT and cKO stimulated splenic CD8^+^ T cells with or without MK-2206 addition. n=3 mice per group (D-G). Statistical significance was determined by one-way ANOVA followed by Tukey's test (D-G). The data are presented as the means ± SD. * P <0.05; **** P<0.0001.

Previous studies have reported that Aldob can directly interact with Akt and inhibit its phosphorylation to limit the metabolism of hepatocellular carcinoma 33. Therefore, we next verified whether Akt signaling is involved in the process of Aldob-mediated regulation of CD8^+^ T cell metabolism. Indeed, Kyoto Encyclopedia of Genes and Genomes (KEGG) analysis based on RNA-sequencing data from WT and cKO CD8^+^ T cells in tumor revealed that the PI3K-Akt pathways accounted for the largest number of altered genes in the enriched pathways (Figure 6A). Similar to reported findings in hepatocellular carcinoma cells, the immunoprecipitation assay showed that Aldob interacted with Akt in CD8^+^ T cells in tumors (Figure 6B). In addition, the level of phosphorylated Akt was also increased in cKO CD8^+^ T cells compared to WT CD8^+^ T cells (Figure 6C). Then, to assess whether the suppression of Akt counteracts the enhanced immune response in cKO CD8^+^ T cells, we added the Akt inhibitor MK-2206 (1 μM) *in vitro*. MK-2206 suppressed the secretion of IFN-γ and TNF-α (Figure 6D) and decreased the production of costimulatory molecules and effector cytokines (Figure 6E, F) in both cKO and WT CD8^+^ T cells. The generation of pyruvate and lactate (Figure S9A) as well as metabolites of the Krebs cycle and ATP in CD8^+^ T cells was also significantly reduced after MK-2206 treatment (Figure 6G and Figure S9B), suggesting that impairment of glycolysis and energy synthesis is the intrinsic basis for T cell dysfunction. Taken together, these results demonstrate that Aldob promotes CBX4-mediated CD8^+^ T cell dysfunction by disrupting the phosphorylation of Akt and subsequently activating glycolysis and energy synthesis.

### Knockout of *Cbx4* combining with anti-PD1 therapy reinforce T cell's anti-tumor effects

**Figure 7:**
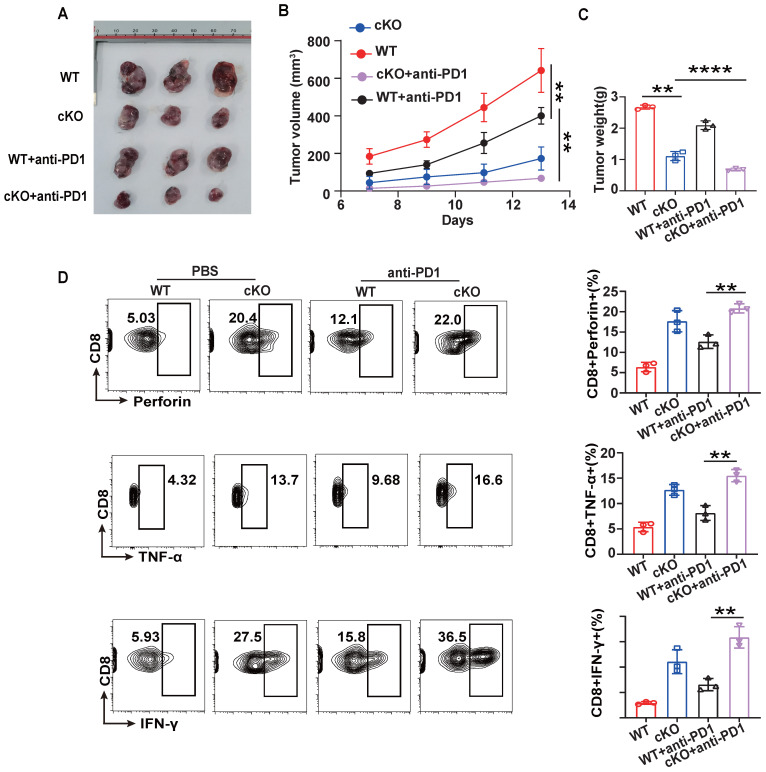
** Knockout of *Cbx4* strengthens the antitumor response of anti-PD1.** (A-C) Analysis of tumor growth, volume and weight in WT and cKO mice challenged with B16F1 cells for 14 days with or without anti-PD1 antibody injection on Days 7, 9 and 11. (D) Flow cytometric analysis results showing the percentages of TNF-α^+^, IFN-γ^+^ and perforin^+^ tumor-derived CD8^+^ T cells from WT and cKO mice with or without anti-PD1 antibody injection. n=3 mice per group (A-D). Statistical significance was determined by one-way ANOVA followed by Tukey's test (A-D). The data are presented as the means ± SD. ** P<0.01; **** P<0.0001.

To further evaluate the potential of CBX4 as an immunotherapeutic target in cancer, we investigated its role in regulating antitumor efficacy in combination with anti-PD1 therapy 34,35. WT or cKO mice bearing B16-F1 tumors were given intraperitoneal injections of an anti-PD1 antibody or PBS on Days 7, 9, and 12. cKO mice combined with anti-PD1 treatment were found to have significantly reduced tumour growth, volume, and weight compared to the anti-PD1 and cKO groups (Figure 7A-C). Furthermore, *Cbx4* knockout in CD8^+^ T cells combined with anti-PD1 treatment significantly increased the production of effector cytokines, including IFN-γ, TNF-α and perforin (Figure 7D). Based on the regulation of cellular metabolism by CBX4, we hypothesized that modulation of metabolism may be key to improving the efficacy of tumor immunotherapy.

## Discussion

Metabolism-mediated energy synthesis and epigenetic regulation directly control T cell activation and function 36. Here, we have shown that CBX4 acts as a checkpoint to inhibit glycolysis and decrease the energy supply through Aldob-mediated downregulation of the *Akt* pathway in CD8^+^ T cells, thereby identifying targeting CBX4 as a potential approach to enhance antitumor immunity.

As a member of the Polycomb protein family, CBX4 has been reported to participate in the formation of cPRC1 and to bind H3K27me3, thereby maintaining the inactive state of chromatin and facilitating the maintenance and self-renewal of pluripotent and multipotent stem cells 37,38. In addition to its known functions in transcriptional repression, CBX4 also regulates protein stability via its sumoylation activity, a function distinct from those of other members of the chromobox protein family 39. For instance, CBX4-mediated sumoylation of PR domain containing 16 (Prdm16) at lysine 917 blocks its ubiquitination-mediated degradation, thereby augmenting its stability and thermogenic function 40. While in another report, CBX4 has also been shown to be able to affect the sumoylation modification of DNA methylase, thereby reacting on the epigenetic modification 41. In our study, the ChIP-Seq assay showed that the CBX4 binding motifs were similar to those of the transcription factors KLF3 and SP1 (Figure 3A, B) and that CBX4 interacts with KLF3 and SP1 to regulate the expression of the metabolism-related molecule Aldob (Figure 4D-G). Consistently, we found that CBX4 promoted the stability and functions of KLF3 and SP1 by modulating their sumoylation, and these effects might be closely associated with the formation of membraneless nuclear structures dependent on sumoylation 42. It has been reported that *Klf2* is highly expressed in quiescent T cells and reduced to undetectable levels upon T cell activation, which is consistent with *Cbx4*, while recent study also revealed that clusters of terminal exhausted T cells characterised with NK cell phenotype is enriched with Sp and Klf subfamily TFs 43,44. Considering these findings collectively with our results led us to speculate that CBX4 may work together with Sp and Klf family TFs via sumoylation to form a transcriptional regulatory axis, thus inhibiting the function and activation of T cells. It is noteworthy that this paradigm, in which CBX4 sumoylates other TFs to maintain the quiescent phenotype of CD8^+^ T cells via TF-mediated transcriptional regulation, deviates from the known epigenetic repression role of CBX4.

Upon antigen stimulation, CD8^+^ T cells exit quiescence and undergo a switch to effector differentiation, while emerging research highlights the critical role of metabolism in these processes 45,46. It is known that the increased demand for biosynthetic intermediates and energy is met by metabolic adaptation via a switch from OXPHOS to aerobic glycolysis 47,48. Aldolase involved in fructose metabolism has been reported to negatively regulate AMP-activated protein kinase (AMPK), implying that Aldob may act as similar glycolysis inhibitor 49. In this study, we found that Aldob, which reversibly catalyzes the conversion of F1,6P to G3P and DHAP, was transcriptionally regulated by the CBX4-associated TFs KLF3 and SP1. Aldob downregulates T cell glycolysis and energy metabolism by inhibiting Akt phosphorylation. Aldob-mediated metabolism, which was indirectly modulated by CBX4, suppressed T cell activation, and led to a state of exhaustion.

In conclusion, our study showed that knocking out *Cbx4* promotes CD8^+^ T cell-mediated antitumor immunity and benefits tumor immunotherapy. Mechanistically, CBX4 promotes the stability of KLF3 and SP1 via their sumoylation, which in turn increases the transcription of the metabolism-associated molecule Aldob. The regulation of Aldob by CBX4 interferes with intracellular glycolysis and subsequent energy production, which makes CBX4 an intrinsic checkpoint for T cell activation. This mechanism increases our understanding of the correlation between metabolism and T cell function, offering a promising new strategy for tumor immunotherapy in the future.

## Supplementary Material

Supplementary figures.

## References

[1] Schurich A, Pallett LJ, Jajbhay D, Wijngaarden J, Otano I, Gill US (2016). Distinct Metabolic Requirements of Exhausted and Functional Virus-Specific CD8 T Cells in the Same Host. Cell Rep.

[2] Chang CH, Pearce EL (2016). Emerging concepts of T cell metabolism as a target of immunotherapy. Nat Immunol.

[3] Palmer CS, Ostrowski M, Balderson B, Christian N, Crowe SM (2015). Glucose metabolism regulates T cell activation, differentiation, and functions. Front Immunol.

[4] Wherry EJ, Kurachi M (2015). Molecular and cellular insights into T cell exhaustion. Nat Rev Immunol.

[5] Sears JD, Waldron KJ, Wei J, Chang CH (2021). Targeting metabolism to reverse T-cell exhaustion in chronic viral infections. Immunology.

[6] Bengsch B, Johnson AL, Kurachi M, Odorizzi PM, Pauken KE, Attanasio J (2016). Bioenergetic Insufficiencies Due to Metabolic Alterations Regulated by the Inhibitory Receptor PD-1 Are an Early Driver of CD8(+) T Cell Exhaustion. Immunity.

[7] Cao X, Li W, Yu Y, Liu T, Zhou Y (2022). China enters CAR-T cell therapy era. Innovation (Camb).

[8] Zhang E, Ma Z, Li Q, Yan H, Liu J, Wu W (2019). TLR2 Stimulation Increases Cellular Metabolism in CD8(+) T Cells and Thereby Enhances CD8(+) T Cell Activation, Function, and Antiviral Activity. J Immunol.

[9] Wang Z, Fang Z, Chen G, Liu B, Xu J, Li F (2021). Chromobox 4 facilitates tumorigenesis of lung adenocarcinoma through the Wnt/beta-catenin pathway. Neoplasia.

[10] Di Croce L, Helin K (2013). Transcriptional regulation by Polycomb group proteins. Nat Struct Mol Biol.

[11] Kim JJ, Kingston RE (2022). Context-specific Polycomb mechanisms in development. Nat Rev Genet.

[12] Parreno V, Martinez AM, Cavalli G (2022). Mechanisms of Polycomb group protein function in cancer. Cell Res.

[13] Mardaryev AN, Liu B, Rapisarda V, Poterlowicz K, Malashchuk I, Rudolf J (2016). Cbx4 maintains the epithelial lineage identity and cell proliferation in the developing stratified epithelium. J Cell Biol.

[14] Hao L, Midic U, Garriga J, Latham KE (2014). Contribution of CBX4 to cumulus oophorus cell phenotype in mice and attendant effects in cumulus cell cloned embryos. Physiol Genomics.

[15] Wang B, Tang J, Liao D, Wang G, Zhang M, Sang Y (2013). Chromobox homolog 4 is correlated with prognosis and tumor cell growth in hepatocellular carcinoma. Ann Surg Oncol.

[16] Chen F, Hou W, Yu X, Wu J, Li Z, Xu J (2023). CBX4 deletion promotes tumorigenesis under Kras(G12D) background by inducing genomic instability. Signal Transduct Target Ther.

[17] Park PJ (2009). ChIP-seq: advantages and challenges of a maturing technology. Nat Rev Genet.

[18] Kim D, Langmead B, Salzberg SL (2015). HISAT: a fast spliced aligner with low memory requirements. Nat Methods.

[19] Love MI, Huber W, Anders S (2014). Moderated estimation of fold change and dispersion for RNA-seq data with DESeq2. Genome Biol.

[20] Pertea M, Pertea GM, Antonescu CM, Chang TC, Mendell JT, Salzberg SL (2015). StringTie enables improved reconstruction of a transcriptome from RNA-seq reads. Nat Biotechnol.

[21] Zhu GQ, Tang Z, Huang R, Qu WF, Fang Y, Yang R (2023). CD36(+) cancer-associated fibroblasts provide immunosuppressive microenvironment for hepatocellular carcinoma via secretion of macrophage migration inhibitory factor. Cell Discov.

[22] Satija R, Farrell JA, Gennert D, Schier AF, Regev A (2015). Spatial reconstruction of single-cell gene expression data. Nat Biotechnol.

[23] Stuart T, Butler A, Hoffman P, Hafemeister C, Papalexi E, Mauck WR (2019). Comprehensive Integration of Single-Cell Data. Cell.

[24] Ren X, Hu B, Song M, Ding Z, Dang Y, Liu Z (2019). Maintenance of Nucleolar Homeostasis by CBX4 Alleviates Senescence and Osteoarthritis. Cell Rep.

[25] Legut M, Gajic Z, Guarino M, Daniloski Z, Rahman JA, Xue X (2022). A genome-scale screen for synthetic drivers of T cell proliferation. Nature.

[26] Russ BE, Olshanksy M, Smallwood HS, Li J, Denton AE, Prier JE (2014). Distinct epigenetic signatures delineate transcriptional programs during virus-specific CD8(+) T cell differentiation. Immunity.

[27] Belk JA, Daniel B, Satpathy AT (2022). Epigenetic regulation of T cell exhaustion. Nat Immunol.

[28] Ren L, Li Z, Zhou Y, Zhang J, Zhao Z, Wu Z (2023). CBX4 promotes antitumor immunity by suppressing Pdcd1 expression in T cells. Mol Oncol.

[29] Wu L, Pan T, Zhou M, Chen T, Wu S, Lv X (2022). CBX4 contributes to HIV-1 latency by forming phase-separated nuclear bodies and SUMOylating EZH2. Embo Rep.

[30] Hay N (2016). Reprogramming glucose metabolism in cancer: can it be exploited for cancer therapy?. Nat Rev Cancer.

[31] Chang CH, Curtis JD, Maggi LJ, Faubert B, Villarino AV, O'Sullivan D (2013). Posttranscriptional control of T cell effector function by aerobic glycolysis. Cell.

[32] Wiley CD, Campisi J (2021). The metabolic roots of senescence: mechanisms and opportunities for intervention. Nat Metab.

[33] He X, Li M, Yu H, Liu G, Wang N, Yin C (2020). Loss of hepatic aldolase B activates Akt and promotes hepatocellular carcinogenesis by destabilizing the Aldob/Akt/PP2A protein complex. Plos Biol.

[34] Hudson WH, Gensheimer J, Hashimoto M, Wieland A, Valanparambil RM, Li P (2019). Proliferating Transitory T Cells with an Effector-like Transcriptional Signature Emerge from PD-1(+) Stem-like CD8(+) T Cells during Chronic Infection. Immunity.

[35] Wang DR, Wu XL, Sun YL (2022). Therapeutic targets and biomarkers of tumor immunotherapy: response versus non-response. Signal Transduct Target Ther.

[36] Liu Q, Zhu F, Liu X, Lu Y, Yao K, Tian N (2022). Non-oxidative pentose phosphate pathway controls regulatory T cell function by integrating metabolism and epigenetics. Nat Metab.

[37] Vandamme J, Volkel P, Rosnoblet C, Le Faou P, Angrand PO (2011). Interaction proteomics analysis of polycomb proteins defines distinct PRC1 complexes in mammalian cells. Mol Cell Proteomics.

[38] Wang S, C OS, Dhiman A, Jiao G, Strohmier BP, Krusemark CJ (2021). Polycomb group proteins in cancer: multifaceted functions and strategies for modulation. NAR Cancer.

[39] Eissenberg JC (2012). Structural biology of the chromodomain: form and function. Gene.

[40] Chen Q, Huang L, Pan D, Zhu LJ, Wang YX (2018). Cbx4 Sumoylates Prdm16 to Regulate Adipose Tissue Thermogenesis. Cell Rep.

[41] Li B, Zhou J, Liu P, Hu J, Jin H, Shimono Y (2007). Polycomb protein Cbx4 promotes SUMO modification of de novo DNA methyltransferase Dnmt3a. Biochem J.

[42] Hickey CM, Wilson NR, Hochstrasser M (2012). Function and regulation of SUMO proteases. Nat Rev Mol Cell Biol.

[43] Cao Z, Sun X, Icli B, Wara AK, Feinberg MW (2010). Role of Kruppel-like factors in leukocyte development, function, and disease. Blood.

[44] Giles JR, Ngiow SF, Manne S, Baxter AE, Khan O, Wang P (2022). Shared and distinct biological circuits in effector, memory and exhausted CD8(+) T cells revealed by temporal single-cell transcriptomics and epigenetics. Nat Immunol.

[45] Hu Z, Qu G, Yu X, Jiang H, Teng XL, Ding L (2019). Acylglycerol Kinase Maintains Metabolic State and Immune Responses of CD8(+) T Cells. Cell Metab.

[46] Rivadeneira DB, DePeaux K, Wang Y, Kulkarni A, Tabib T, Menk AV (2019). Oncolytic Viruses Engineered to Enforce Leptin Expression Reprogram Tumor-Infiltrating T Cell Metabolism and Promote Tumor Clearance. Immunity.

[47] Orozco JM, Krawczyk PA, Scaria SM, Cangelosi AL, Chan SH, Kunchok T (2020). Dihydroxyacetone phosphate signals glucose availability to mTORC1. Nat Metab.

[48] Belikov AV, Schraven B, Simeoni L (2015). T cells and reactive oxygen species. J Biomed Sci.

[49] Zhang CS, Hawley SA, Zong Y, Li M, Wang Z, Gray A (2017). Fructose-1,6-bisphosphate and aldolase mediate glucose sensing by AMPK. Nature.

