# Supplementary material for: CBX4 suppresses CD8+ T cell antitumor immunity by reprogramming glycolytic metabolism
Source: Theranostics. 2024 Jun 17;14(10):3793–809. doi: 10.7150/thno.95748 (PMC11234269; doi:10.7150/thno.95748)
Supplement: Supplementary file 1 — Supplementary figures. [file thnov14p3793s1.pdf]

Supplementary Data

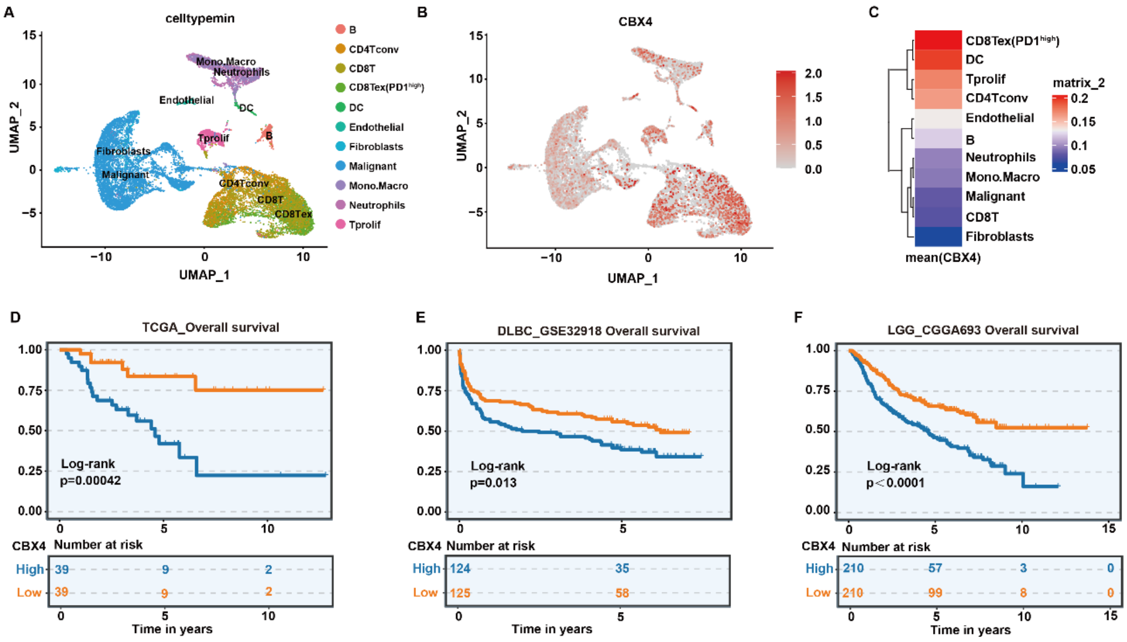

Fig. S1: High expression of *Cbx4* promotes tumor development in humans

(A-C) Single-cell sequencing analysis of cells from 5 patients with anaplastic thyroid cancer (ATC) (GSE148673). (A) Uniform manifold approximation and projection (UMAP) plot of immune-associated cells according to cluster classification. (B) Normalized gene expression of *Cbx4* projected onto the UMAP embedding. (C) Heatmap showing the expression of *Cbx4* in all identified cluster signatures. (D) Survival analysis of 39 adrenal cortical carcinoma (ACC) patients with high or low expression of *Cbx4* (TCGA). (E) Survival analysis of 125 diffuse large B-cell lymphoma (DLBC) patients with high or low expression of *Cbx4* (GSE32918). (F) Survival analysis of 210 low-grade glioma (LGG) patients with high or low expression of *Cbx4* (CGGA693).

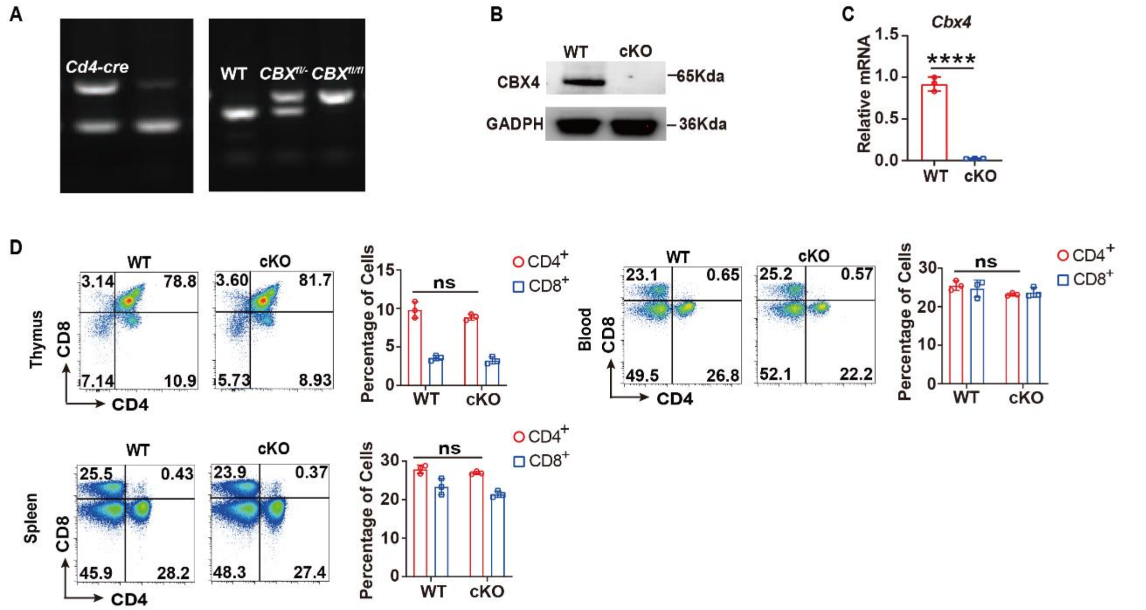

Fig. S2: *Cbx4* knockout didn't interfere with T cells' differentiation and development.

(A) Genotyping results of *Cbx4*$^{fl/fl}$*Cd4-cre* (cKO) mice. (B) Immunoblot analysis of CBX4 in splenic T cells from WT and cKO mice. (C) RT-PCR analysis of CBX4 in splenic T cells from WT and cKO mice. (D) Proportional analysis of Thymus, Blood, Spleen CD4$^+$ and CD8$^+$ T cells in cKO and WT mice. n=3 per group (C, D). Statistical significance was determined by two-tailed unpaired Student's t test (E). The data are presented as the means ± SEM. **** P<0.0001. ns represents no statistical significance.

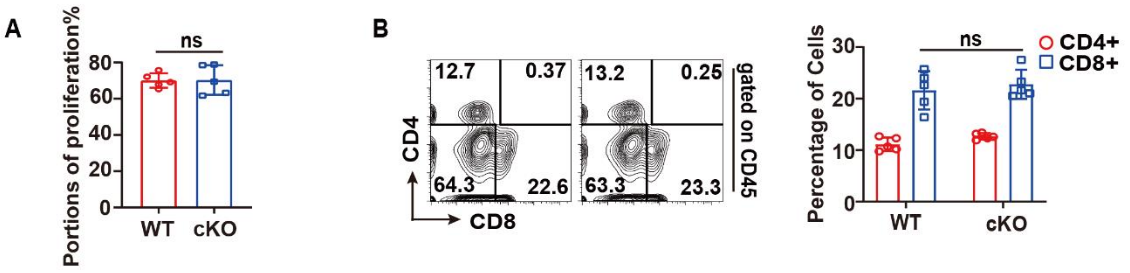

Fig. S3: *Cbx4* knockout didn't interfere with T cells' proliferation.

(A) Proportional analysis of proliferous CD8+ T cells after CD3/28 stimulation for 3 days. (B) Proportional analysis of tumor CD4+ and CD8+ T cells. n=3 mice per group (A); n=6 mice per group (B). Statistical significance was determined by two-tailed unpaired Student's t test (A, B). The data are from three independent experiments (A, B). The data are presented as the means ± SEM. ns represents no statistical significance.

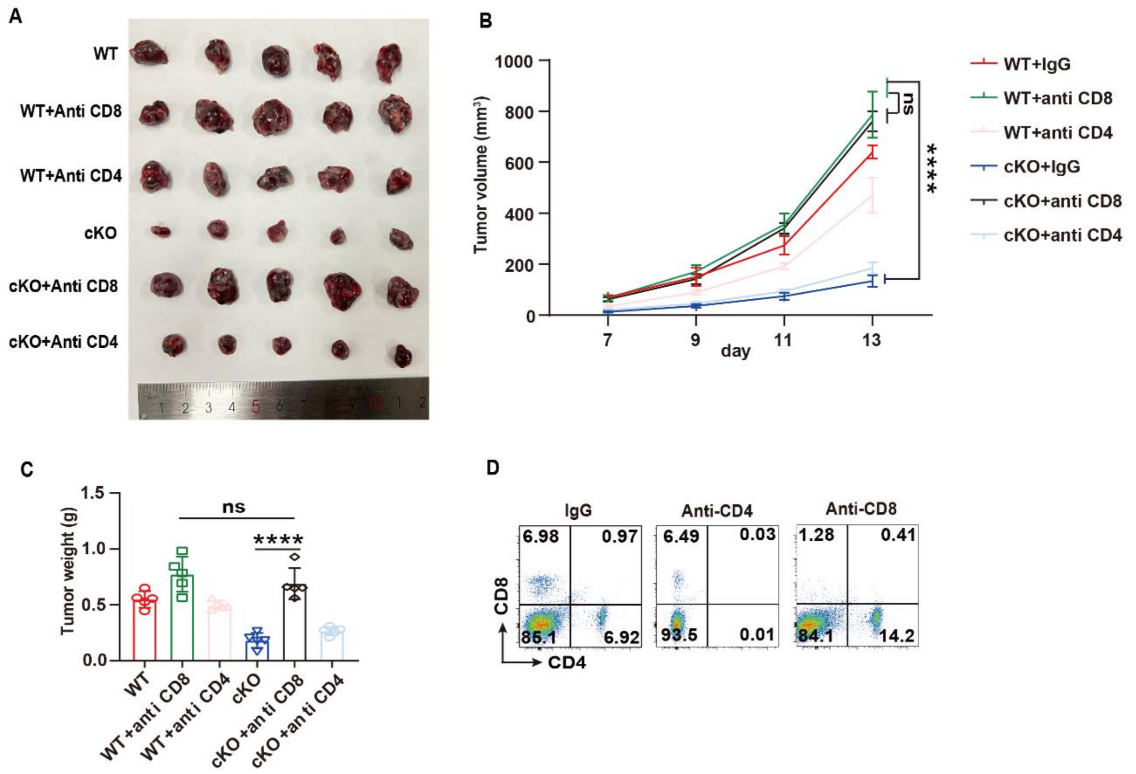

Fig. S4: *Cbx4* knockout CD8+ T cells results in higher anti-tumor responses.

(A-C) B16F1 tumor growth (A), volume(B) and weight (C) in cKO and WT mice on Day 14 after cell inoculation with or without anti-CD4/CD8 treatment. (D) CD4+ and CD8+ portions of spleen lymphocytes with or without anti-CD4/CD8 treatment. n=5 mice per group (A, B and C). Statistical significance was determined by two-tailed unpaired Student's t test (B, C). The data are

representative of three independent experiments (A-C). The data are presented as the means ± SEM. **** P<0.0001.

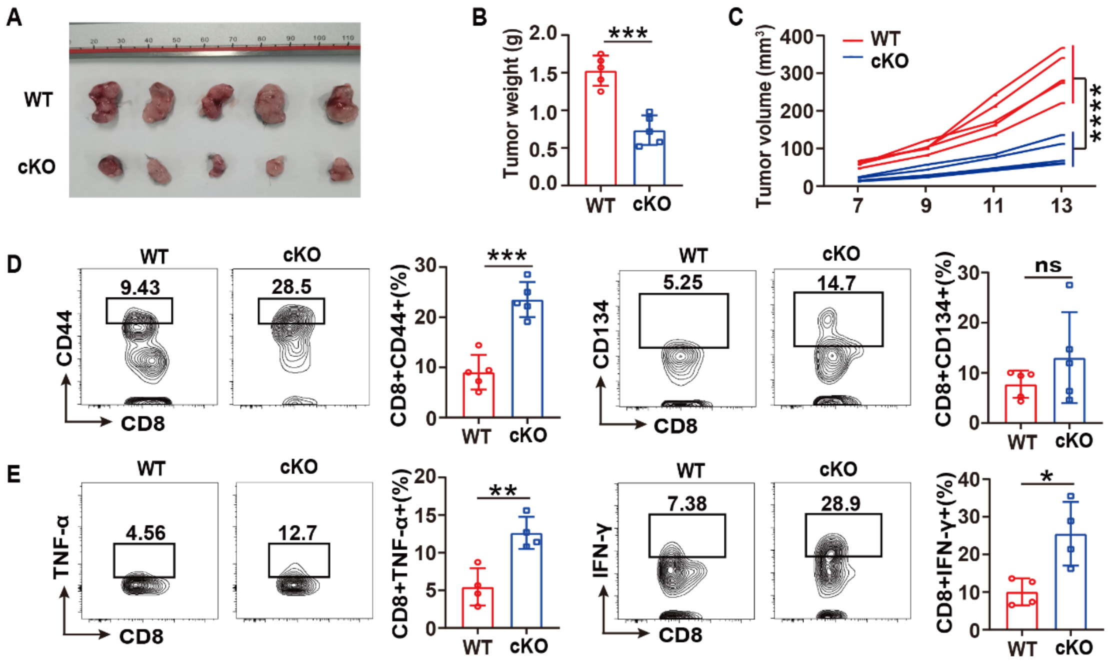

Fig. S5: *Cbx4* knockout T cells exhibit enhanced antitumor function in Hep1-6 tumor models

(A-C) Hep1-6 tumor growth (A), weight (B) and volume (C) in cKO and WT mice on Day 14 after cell inoculation. (D-E) Flow cytometric analysis results showing the percentages of $CD44^+$ and $CD134^+$ (D) and TNF-α^+ and IFN-γ^+ (E) $CD8^+$ T cells in subcutaneous Hep1-6 tumors of WT or cKO mice 14 days after tumor cell inoculation. n=5 mice per group (A, B and C); n=5 mice per group (D); n=4 mice per group (E). The data are representative of two independent experiments (A-E). Statistical significance was determined by two-tailed unpaired Student's t test (A-E). The data are presented as the means ± SEM. * P <0.05; ** P<0.01; *** P<0.001; ns means no statistical significance.

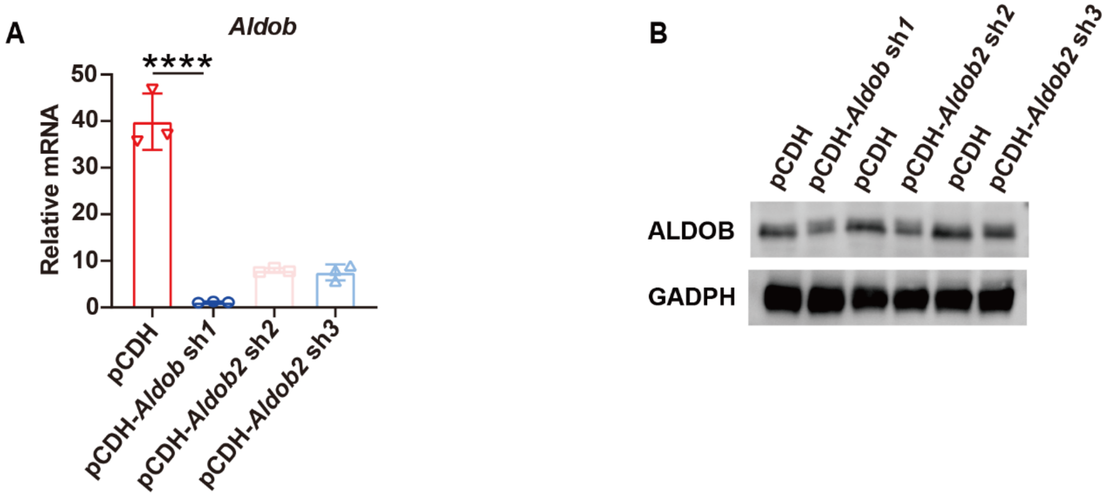

Fig. S6: Knockout of *Cbx4* in CD8$^+$ T cells did not influence chromatin accessibility

(A) Chromatin accessibility analysis of WT and cKO tumor CD8$^+$ T cells. (B) Chromatin accessibility analysis of gene loci of *Pdcd1*. (C) Motif analysis based on the CBX4 binding nucleotide sequence.

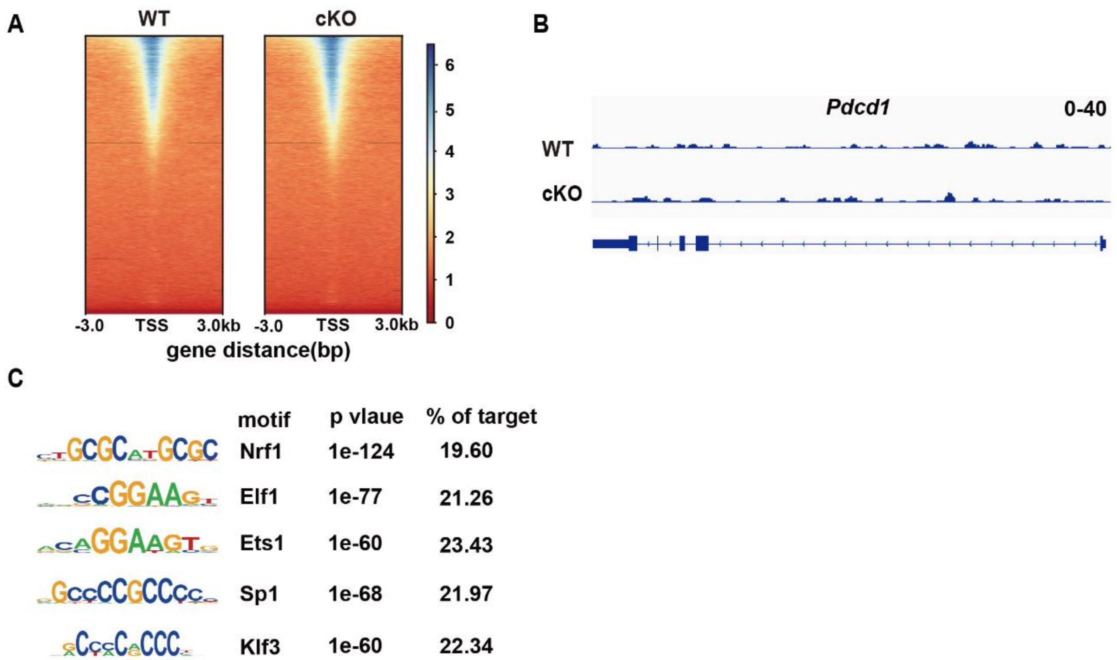

Fig. S7: Identification of sh-*Aldob* in CD8$^+$ T cells.

(A)Verification of Sh-*Aldob* lentivirus in CD8$^+$ T cells based on qPCR experiments. (B) Verification of Sh-*Aldob* lentivirus in CD8$^+$ T cells based on immunoblots experiments. n=3 mice per group (A). The data are representative of two independent experiments (A). Statistical significance was determined by two-tailed unpaired Student's t test (A). The data are presented as the means ± SEM. **** P <0.0001;

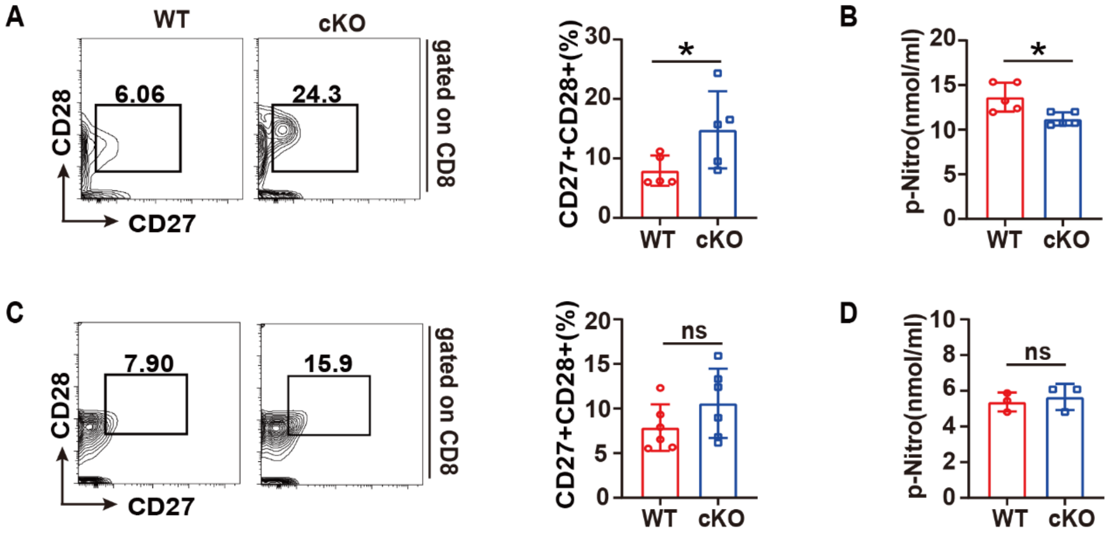

Fig. S8: CBX4 promotes senescence through metabolic regulation.

(A, C) Flow cytometric analysis results showing the percentages of CD27$^+$CD28$^+$ cells among tumor-derived CD8$^+$ T cells (A) and stimulated splenic CD8$^+$ T cells (C). (B, D) Measurement of the β-gal concentration in tumor-derived CD8$^+$ T cells (B) and stimulated splenic CD8$^+$ T cells. (D). n=5 mice per group (A, B). n=6 mice per group (C). n=3 mice per group (D). Statistical significance was determined by two-tailed unpaired Student's t test (A-D). The data are presented as the means ± SD. * P <0.05; ns means not statistically significant.

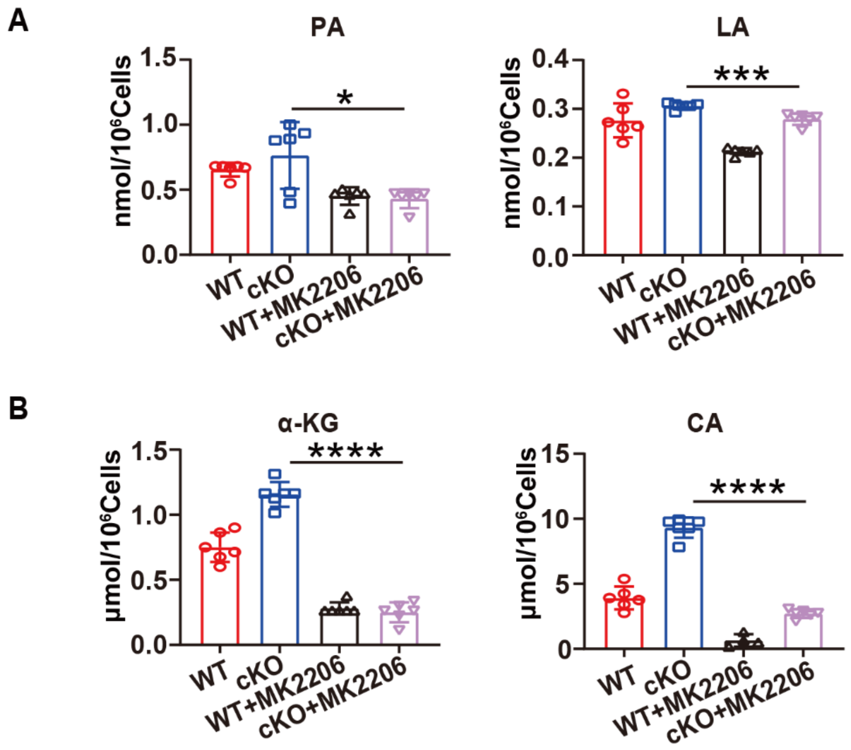

Fig. S9: Inhibition of Akt counteracts the enhanced glycolysis and energy production in cKO CD8$^+$ T cells.

(A) Measurement of pyruvate and lactate levels in WT and cKO stimulated splenic CD8$^+$ T cells with or without MK-2206 treatment. (B) α-Ketoglutarate and citrate levels in stimulated splenic CD8$^+$ T cells with or without MK-2206 treatment. n=3 mice per group (A, B). Statistical significance was determined by one-way ANOVA followed by Tukey's test (A, B). PA, pyruvate; LA, lactate; α-KG, α-ketoglutarate; CA, citrate. * $P < 0.05$; ** $P<0.01$; *** $P<0.001$; **** $P<0.0001$.
